# An Organic Borate Salt with Superior *p*‐Doping Capability for Organic Semiconductors

**DOI:** 10.1002/advs.202001322

**Published:** 2020-07-06

**Authors:** Berthold Wegner, Dominique Lungwitz, Ahmed E. Mansour, Claudia E. Tait, Naoki Tanaka, Tianshu Zhai, Steffen Duhm, Michael Forster, Jan Behrends, Yoshiaki Shoji, Andreas Opitz, Ullrich Scherf, Emil J. W. List‐Kratochvil, Takanori Fukushima, Norbert Koch

**Affiliations:** ^1^ Institut für Physik and IRIS Adlershof Humboldt‐Universität zu Berlin Berlin D‐12489 Germany; ^2^ Helmholtz‐Zentrum Berlin für Materialien und Energie GmbH Berlin D‐12489 Germany; ^3^ Berlin Joint EPR Lab Fachbereich Physik Freie Universität Berlin Berlin D‐14195 Germany; ^4^ Laboratory for Chemistry and Life Science Institute of Innovative Research Tokyo Institute of Technology Yokohama 226‐8503 Japan; ^5^ Institute of Functional Nano and Soft Materials (FUNSOM) Jiangsu Key Laboratory for Carbon‐Based Functional Materials and Devices and Joint International Research Laboratory of Carbon‐Based Functional Materials and Devices Soochow University Suzhou 215123 P. R. China; ^6^ Makromolekulare Chemie and Institut für Polymertechnologie Bergische Universität Wuppertal Wuppertal D‐42097 Germany; ^7^ Institut für Chemie Humboldt‐Universität zu Berlin Berlin D‐12489 Germany

**Keywords:** bipolarons, borate salt, doping, organic semiconductors, polarons

## Abstract

Molecular doping allows enhancement and precise control of electrical properties of organic semiconductors, and is thus of central technological relevance for organic (opto‐) electronics. Beyond single‐component molecular electron acceptors and donors, organic salts have recently emerged as a promising class of dopants. However, the pertinent fundamental understanding of doping mechanisms and doping capabilities is limited. Here, the unique capabilities of the salt consisting of a borinium cation (Mes_2_B^+^; Mes: mesitylene) and the tetrakis(penta‐fluorophenyl)borate anion [B(C_6_F_5_)_4_]^−^ is demonstrated as p‐type dopant for polymer semiconductors. With a range of experimental methods, the doping mechanism is identified to comprise electron transfer from the polymer to Mes_2_B^+^, and the positive charge on the polymer is stabilized by [B(C_6_F_5_)_4_]^−^. Notably, the former salt cation leaves during processing and is not present in films. The anion [B(C_6_F_5_)_4_]^−^ even enables the stabilization of polarons and bipolarons in poly(3‐hexylthiophene), not yet achieved with other molecular dopants. From doping studies with high ionization energy polymer semiconductors, the effective electron affinity of Mes_2_B^+^[B(C_6_F_5_)_4_]^−^ is estimated to be an impressive 5.9 eV. This significantly extends the parameter space for doping of polymer semiconductors.

## Graphical abstract

The salt of the cation (Mes_2_B^+^; Mes: mesitylene) and the anion [B(C_6_F_5_)_4_]^−^ is introduced as superior p‐type dopant for organic semiconductors. The doping mechanism involves electron transfer from the semiconductor to Mes_2_B^+^, and the positive charge is stabilized by [B(C_6_F_5_)_4_]^−^. For poly(3‐hexylthiophene), the anion even stabilizes bipolarons. The effective electron affinity of Mes_2_B^+^[B(C_6_F_5_)_4_]^−^ is estimated to be 5.9 eV.

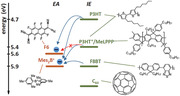

## 1 Introduction

Doping is the process of introducing a small amount of dopants (atoms or molecules) to a semiconductor in order to tune its electrical properties, such as Fermi level position, charge carrier density, and carrier mobility. The ability of controlled doping was a breakthrough for inorganic semiconductors, because it allowed precise control of charge transport and the creation of p–n‐junctions, both of which were key to the development of novel device concepts, like the bipolar transistor, and thus present semiconductor technology. In an analogous manner, the concept of doping was extended to organic semiconductors. Here, the use of molecular strong electron donors or acceptors as dopants has emerged as the most viable approach, which is often termed “electrical doping” or “chemical doping.”^[^ ^1^, ^2^, ^3^, ^4^ ^]^ Molecular dopants were employed to enhance the performance of organic light emitting diodes (OLEDs),^[^ ^5^, ^6^ ^]^ organic photovoltaic cells (OPVCs),^[^ ^7^, ^8^ ^]^ and organic field effect transistors (OFETs).^[^ ^9^, ^10^, ^11^ ^]^ These doping strategies were recently instrumental for achieving high hole^[^ ^12^ ^]^ and electron^[^ ^13^ ^]^ mobility in transistors, enabling coherent charge transport in polymers,^[^ ^14^ ^]^ boosting the power conversion efficiency in organic solar cells,^[^ ^15^ ^]^ and demonstrating high‐performance thermoelectric organic materials.^[^ ^16^ ^]^ The nature of charge carriers formed upon doping conjugated polymers with a nondegenerate ground state, like the most widely studied poly(3‐hexylthiophene) (P3HT), has been a widely discussed topic. While many studies favored the occurrence of mainly positive polarons (cation segment on a polymer chain), others suggested the formation of mainly positive bipolarons (dication segment on a polymer chain).^[^ ^17^, ^18^, ^19^, ^20^, ^21^, ^22^, ^23^ ^]^ Today, it is commonly accepted that molecular doping of polymers, in general, leads to the formation of polarons as charge carriers, predominantly.^[^ ^1^, ^2^, ^24^, ^25^ ^]^ Positive bipolaron formation in P3HT has been evidenced only upon electrochemical doping and doping with FeCl_3_ (a small inorganic Lewis acid dopant),^[^ ^26^, ^27^, ^28^, ^29^ ^]^ but not with the molecular dopants employed nowadays, such as 2,3,5,6‐tetrafluoro‐7,7,8,8‐tetracyanoquinodimethane (F_4_TCNQ),^[^ ^4^, ^30^, ^31^, ^32^ ^]^ 1,3,4,5,7,8‐hexafluoro‐11,11,12,12‐tetracyanonaphtho‐2,6‐quinodimethane (F_6_TCNNQ),^[^ ^33^ ^]^ hexacyano‐trimethylene‐cyclopropane (CN6‐CP),^[^ ^34^ ^]^ and molybdenum tris[1,2‐bis(trifluoromethyl)ethane‐1,2‐dithiolene] (Mo(tfd)_3_).^[^ ^35^, ^36^ ^]^ While bipolaron formation was considered to occur in some of these studies,^[^ ^22^, ^37^ ^]^ no compelling evidence was provided, particularly as no clear differentiation of polaron and bipolaron abundance in dependence of dopant concentration was possible. This raises the question whether these dopants are not sufficiently strong to support bipolaron formation, or whether other factors apparently inhibit this process, which for electrochemical doping and doping with FeCl_3_ seems possible.

More recently, novel dopants based on organic salts have emerged,^[^ ^16^, ^38^, ^39^, ^40^, ^41^ ^]^ providing additional possibilities for doping procedures, beyond the neutral single‐component molecular dopants mentioned above. Despite the opportunities offered by such doping approaches, comparably little is known about the fundamental doping processes, the expected charge‐exchange reactions, and, notably, the fate in thin films of that salt ion, which is not further needed once doping has occurred, and thus the overall charge balance.

**Figure 1:**
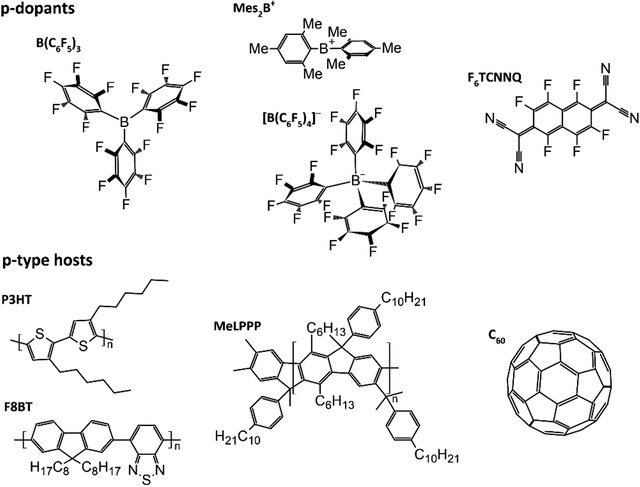
Chemical structure of the compounds used in this study. Poly(3‐hexylthiophene‐2,5‐diyl) (P3HT), ladder‐type methylated poly(*para*‐phenylene) (MeLPPP), poly[(9,9‐dioctylfluorenyl‐2,7‐diyl)‐*co*‐(1,4‐benzo‐{2,1′,3}thiadiazole)] (F8BT), C_60_, as well as the three dopants tris(pentafluorophenyl)borane [B(C_6_F_5_)_3_], the organic salt consisting of a diarylborinium ion (Mes_2_B^+^, Mes = mesityl) and a tetrakis(pentafluorophenyl)borate anion ([B(C_6_F_5_)_4_]^−^), and hexafluoro‐tetracyano‐naphthoquinodimethane (F_6_TCNNQ).

Here, we investigate the fundamental doping processes involved upon adding an organic salt, consisting of a two‐coordinate borinium cation (Mes_2_B^+^; Mes: mesitylene) and the weakly coordinating tetrakis(penta‐fluorophenyl)borate anion ([B(C_6_F_5_)_4_]^−^),^[^ ^44^ ^]^ to semiconducting polymers. We first compare the p‐doping phenomenology of P3HT with Mes_2_B^+^[B(C_6_F_5_)_4_]^−^ with that of the Lewis acid based dopant tris(pentafluorophenyl)borane [B(C_6_F_5_)_3_]. ^[^ ^44^, ^45^, ^46^, ^47^ ^]^ The chemical structures of all compounds are depicted in **Figure** **1**. Recently, the doping mechanism of B(C_6_F_5_)_3_ has been suggested to result from the formation of the known water‐Lewis acid complex,^[^ ^48^ ^]^ which is a source of H^+^, leading to a protonation of the polymer backbone, and in turn doping a neutral segment of the polymer.^[^ ^49^ ^]^ In contrast, the organic salt Mes_2_B^+^[B(C_6_F_5_)_4_]^−^ can serve as a strong one‐electron oxidant due to the exceptionally electron‐deficient nature of Mes_2_B^+^.^[^ ^50^, ^51^, ^52^ ^]^ The chemically inert and thermally stable counterion [B(C_6_F_5_)_4_]^−^ was recently also used in combination with the triphenylmethyl cation to p‐dope different polymers and enhance the p‐channel performance of OFETs with doped polymer layers.^[^ ^40^ ^]^ We find that B(C_6_F_5_)_3_ doping results only in polaron formation in P3HT, as for other molecular dopants so far. In contrast, we compile compelling evidence that Mes_2_B^+^[B(C_6_F_5_)_4_]^−^ supports the formation of bipolarons in P3HT, already at intermediate dopant loading. By exploring the doping capability of the salt with high ionization energy (IE) semiconductor polymers, i.e., methylated poly(*para*‐phenylene) (MeLPPP) and the donor–acceptor polymer poly(9,9‐dioctylfluorene‐alt‐benzothiadiazole) (F8BT), we derive a very high effective electron affinity (EA) of Mes_2_B^+^[B(C_6_F_5_)_4_]^−^ of ≈5.9 eV. Furthermore, the doping mechanism is identified to comprise electron transfer from the semiconductor polymer to Mes_2_B^+^, which leaves as volatile compound during film fabrication, and only the p‐doped polymer with [B(C_6_F_5_)_4_]^−^ as charge stabilizing agent for polarons/bipolarons remain in the films, according to

(1) $$\begin{array}{ccc} \mathrm{polyme}r^{0}+\mathrm{Me}s_{2}B^{+}{\left[B{\left(C_{6}F_{5}\right)}_{4}\right]}^{-} & \to & \mathrm{polyme}r^{+}+{\left[B{\left(C_{6}F_{5}\right)}_{4}\right]}^{-}\\ & + & \mathrm{Me}s_{2}B^{•}↑ \end{array}$$

## 2 Results and Discussion

### 2.1 Indications for Bipolarons from Optical Absorption and Conductivity

**Figure 2:**
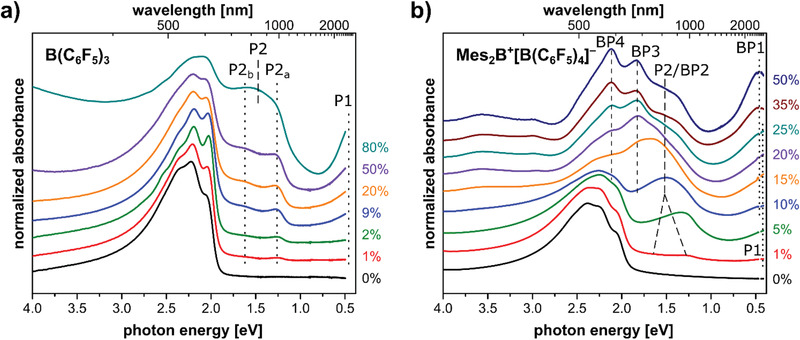
Optical absorption spectra of P3HT films for increasing dopant concentration. a) B(C_6_F_5_)_3_ as dopant and b) Mes_2_B^+^[B(C_6_F_5_)_4_]^−^ as dopant. For assignment of the peak labels, see text.

The optical absorption spectra of B(C_6_F_5_)_3_‐ and Mes_2_B^+^[B(C_6_F_5_)_4_]^−^‐doped P3HT thin films are compared in **Figure** **2** for different dopant concentrations. The spectra of the undoped polymer films exhibit the typical optical signatures of regioregular P3HT thin films with crystalline aggregates, indicated by well‐resolved features at 2.1, 2.2, and 2.4 eV.^[^ ^53^, ^54^, ^55^ ^]^ The spectra of B(C_6_F_5_)_3_‐doped P3HT in Figure 2a show the emergence of the P1 and the two P2 transitions of positive P3HT polarons,^[^ ^56^ ^]^ which were reported from experiments in the range of 0.4–0.5 eV (P1) and 1.3–1.6 eV (P2), respectively.^[^ ^22^, ^24^, ^30^, ^31^, ^57^, ^58^ ^]^ Up to 50% dopant concentration, the two polaron features P2_a_ and P2_b_ are well resolved at 1.3 and 1.6 eV, respectively, and grow in intensity along with the P1 transition, whose maximum cannot be seen in this plot, but is at ≈0.4 eV (see full range spectra in Figure S1 in the Supporting Information). For very high nominal dopant concentration (80%), only one broad feature covering the P2 spectral range is observed. Recently, it was clarified that the observation of one P2 feature indicates the presence of weakly interacting individual P3HT chains, whereas it is split into P2_a_ and P2_b_ in P3HT (crystalline) aggregates due to interchain interactions.^[^ ^59^ ^]^ Apparently, with B(C_6_F_5_)_3_ as dopant, P3HT can still form these aggregates in films up to 50% dopant loading (with dopant anions residing in the periphery of aggregates), while aggregation is significantly suppressed for 80% loading. Yet, up to 50% dopant concentration the polaron features do not shift.

For Mes_2_B^+^[B(C_6_F_5_)_4_]^−^‐doped P3HT (Figure 2b) the absorption spectra exhibit also the P1 and P2 transitions, but only up to about 10% dopant concentration. In particular, at 1% the double feature P2_a,b_ is faintly observed, but merges into one broad P2 feature at 5%, indicative of individual P3HT chains (vide supra). For 10% dopant concentration and beyond, the P2 feature merges into P2/BP2 in Figure 2b, and two additional features at 1.8 eV (BP3) and 2.1 eV (BP4) arise and increase in intensity with increasing dopant concentration; such were not observed for B(C_6_F_5_)_3_ as dopant. Furthermore, while the peak maximum of the low energy transition P1 is not within the spectral range of the plot, a clear peak is seen for 35% and 50% dopant concentration, at ≈0.45 eV and labeled as BP1. These observations strongly indicate the formation of species other than the P3HT polaron, and the bipolaron seems to be a natural candidate. However, the absorption spectra evolution observed here for Mes_2_B^+^[B(C_6_F_5_)_4_]^−^‐doped P3HT is unparalleled in literature, not even for FeCl_3_ or electrochemically doped P3HT films, for which bipolaron formation was postulated.^[^ ^26^, ^28^ ^]^ Consequently, further insight to understand the origin of these new absorption features is provided in the following.

**Figure 3:**
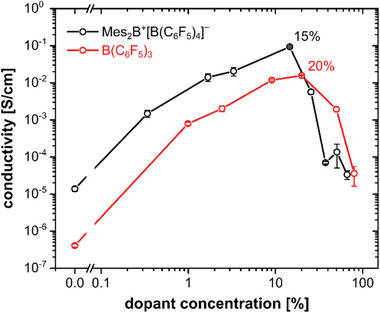
Conductivity of B(C_6_F_5_)_3_‐ and Mes_2_B^+^[B(C_6_F_5_)_4_]^−^‐doped P3HT films. Shown as a function of dopant concentration measured under inert atmosphere. The differences in the conductivity of pristine P3HT are due to two different batches used, as described in the Experimental Section.

The conductivity of P3HT films as function of dopant concentration is given in **Figure** **3**. For both dopants, the conductivity increases by up to five orders of magnitude with increasing dopant concentration, reaching a maximum at around 15% for Mes_2_B^+^[B(C_6_F_5_)_4_]^−^ (0.5 S cm^−1^) and around 20% for B(C_6_F_5_)_3_ (0.02 S cm^−1^), compared to the conductivity of pristine P3HT (4 × 10^−7^ and 1 × 10^−5^ S cm^−1^, respectively, for the two different P3HT batches; see the Experimental Section). We note that mild annealing of Mes_2_B^+^[B(C_6_F_5_)_4_]^−^‐doped P3HT further increases the conductivity (up to 2.8 S cm^−1^; see Figure S2 in the Supporting Information), but beyond 100 °C conductivity drops. The dopant concentration dependent behavior observed here is similar to that for doping of P3HT with F_4_TCNQ, where maximal conductivities of 1–3 S cm^−1^ were achieved at dopant concentrations of around 10–25%.^[^ ^4^, ^32^, ^60^ ^]^ The decrease in conductivity at higher dopant concentration is typically due to a loss in film crystallinity as well as increased Coulomb scattering upon excessive incorporation of dopants. This decreases the charge carrier mobility and thus the conductivity.^[^ ^4^, ^61^ ^]^ However, the decrease in conductivity appears more sudden for P3HT films doped with Mes_2_B^+^[B(C_6_F_5_)_4_]^−^ (in the same dopant concentration range where massive changes in the absorption spectra were observed above) compared to those doped with B(C_6_F_5_)_3_. Therefore, we performed electron paramagnetic resonance (EPR) spectroscopy investigations to obtain insight into the concentration of unpaired spins, and thus charge carrier density and type.

### 2.2 Charge Carrier Density and Type from EPR Measurements

**Figure 4:**
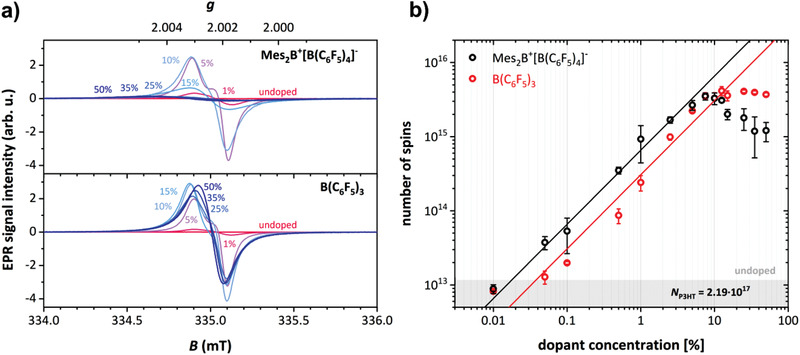
EPR measurements give the unpaired spin density as function of dopant concentration. a) X‐band continuous wave EPR spectra recorded for B(C_6_F_5_)_3_‐ and Mes_2_B^+^[B(C_6_F_5_)_4_]^−^‐doped P3HT films with different dopant concentrations. b) Number of unpaired spins *N* in dependence of the dopant concentration *c* of B(C_6_F_5_)_3_‐ and Mes_2_B^+^[B(C_6_F_5_)_4_]^−^‐doped P3HT films as determined by quantitative EPR measurements. The lines with slope 1 indicate a linear correlation between *N* and *c* in the regime of 0.01% to 5%. The gray area shows the background number of spins measured for pristine P3HT (close to the detection limit). *N* values were determined from three independently performed doping series and the error bars shown correspond to the standard deviation. The number of polymer units *N*_P3HT_ was kept constant at 2.19 × 10^17^ for all samples.

The EPR spectra recorded for P3HT films with different dopant concentrations are shown in **Figure** **4**a. Identical EPR signatures were obtained for both types of dopants at low dopant concentrations and the observed signal can be assigned to the P3HT polaron, based on comparison to literature *g*‐values (see Figure S3 in the Supporting Information).^[^ ^62^ ^]^ The EPR measurements did not show any evidence for the presence of additional dopant‐based paramagnetic species, in line with the fact that the Mes_2_B^+^[B(C_6_F_5_)_4_]^−^ salt cation and anion are diamagnetic, as similarly reported for [B(C_6_F_5_)_4_]^−^ with a trityl cation.^[^ ^16^, ^40^ ^]^ This observation, however, already points toward a doping mechanism that does not yield the Mes_2_B neutral radical, at least not in the films. The determined number of spins, shown in Figure 4b, in the doped samples therefore provides a direct estimate of the number of charge carriers on P3HT. The doping efficiency of B(C_6_F_5_)_3_ and Mes_2_B^+^[B(C_6_F_5_)_4_]^−^ can be estimated from the change in the number of spins as a function of the number of dopant molecules in the linear region, extending up to dopant concentrations of about 5%. This doping efficiency amounts to ≈14% for B(C_6_F_5_)_3_ and ≈30% for Mes_2_B^+^[B(C_6_F_5_)_4_]^−^. The doping efficiency determined for B(C_6_F_5_)_3_ with P3HT is in good agreement with a previous study, where it was also shown that the number of spins determined by EPR matched the mobile hole density determined by admittance spectroscopy.^[^ ^46^ ^]^ At dopant concentrations exceeding 5%, the behavior of B(C_6_F_5_)_3_‐ and Mes_2_B^+^[B(C_6_F_5_)_4_]^−^‐doped films starts to deviate from a linear relationship, showing saturation for B(C_6_F_5_)_3_ and a decrease in spin concentration for Mes_2_B^+^[B(C_6_F_5_)_4_]^−^. This decrease in spin concentration is accompanied by a significant increase in linewidth and a shift to a Lorentzian line shape. Relaxation time measurements confirmed that above dopant concentrations of 10%, the spectral shapes are determined by lifetime broadening with *T*_1_ ≈ *T*_2_ ≤ 50 ns (see Figure S4 in the Supporting Information). Notably, the decrease in spin concentration and broadening of the EPR spectra observed for Mes_2_B^+^[B(C_6_F_5_)_4_]^−^ at high dopant concentrations is correlated with the changes in the absorption spectra (Figure 2) and the measured decrease in conductivity (Figure 3). This is further substantiated by the trend of the resonator *Q*‐factors as function of dopant concentration (shown in Figure S5 in the Supporting Information).

The initial linear increase in spin concentration with dopant concentration is attributed to the increased formation of polarons by doping, in agreement with the observed almost linear increase in conductivity. In the case of B(C_6_F_5_)_3_, as for most dopants investigated so far, no more additional charge carriers are created at high dopant concentrations and, due to the decrease in carrier mobility because of lowered crystallinity, the conductivity decreases. The decrease in spin concentration at high dopant concentrations observed for Mes_2_B^+^[B(C_6_F_5_)_4_]^−^, however, suggests the formation of a spinless (EPR silent) species. Previous EPR investigations on oligothiophenes and thiophene‐based polymers have interpreted similar observations in terms of formation of bipolarons (dications), antiferromagnetically coupled polaron pairs, or interchain polaron dimers (*π*‐dimers), with the type of spinless charge carrier formed being dependent on the polymer conjugation length and nature of the counterion.^[^ ^63^, ^64^, ^65^, ^66^ ^]^ In a recent combined optical, dielectric, and EPR study of iodine vapor‐doped P3HT films, a decrease in spin concentration at high dopant levels, accompanied by significant broadening of the EPR line, was interpreted in terms of formation of antiferromagnetically coupled polaron pairs.^[^ ^67^ ^]^ In that case, however, the optical spectra showed progressive growth of only polaronic features across the whole range of doping levels, accompanied by a slight shift of the highest energy absorption band toward lower energies at doping levels associated with the formation of polaron pairs. The measured conductivity also continued to increase while the spin concentration decreased, which was interpreted in terms of a higher mobility for the polaron pairs compared to polarons. The distinct differences observed in the optical spectra and conductivity measurements in the present doping study thus suggests that the formation of coupled polaron pairs for doping of P3HT with Mes_2_B^+^[B(C_6_F_5_)_4_]^−^ can be excluded. Rather, the clearly observed correlated trends in optical absorption, conductivity, and charge carrier density represent circumstantial evidence for the formation of bipolarons at increased dopant concentration. This is further substantiated by the following analysis of core levels and the valence electronic properties.

### 2.3 Polaron versus Bipolaron Abundance

**Figure 5:**
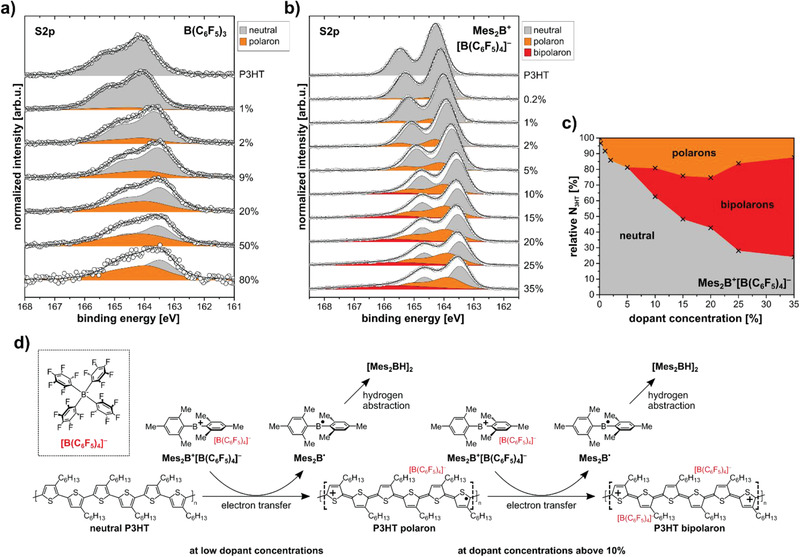
Relative abundance of polarons versus bipolarons in Mes_2_B^+^[B(C_6_F_5_)_4_]^−^‐doped P3HT and suggested doping mechanism. XPS S2p core level spectra with deconvolution into neutral (gray), polaron (orange), and bipolaron (red) signatures for a) B(C_6_F_5_)_3_‐ and b) Mes_2_B^+^[B(C_6_F_5_)_4_]^−^‐doped P3HT. Circles display the raw data. The difference in energy resolution between (a) and (b) stems from the use of two different experimental setups. c) Relative amount of neutral thiophene units (gray), and thiophene units hosting polarons (orange) and bipolarons (red) for Mes_2_B^+^[B(C_6_F_5_)_4_]^−^‐doped P3HT, as explained in the text. d) Proposed doping mechanism of P3HT with Mes_2_B^+^[B(C_6_F_5_)_4_]^−^. First, an electron is transferred from a neutral P3HT segment to Mes_2_B^+^ (leaving as volatile compound during film fabrication), and the formed polaron (cation) on P3HT is stabilized by the anion [B(C_6_F_5_)_4_]^−^. At higher dopant concentrations, the formation of bipolarons is possible. Note: the length of the P3HT segments is chosen for illustrative purposes and does not necessarily reflect the actual charge localization length of polarons and bipolarons.

Further insights into the type of charge carriers generated by doping are obtained by analysis of the sulfur core level spectra as measured by X‐ray photoemission spectroscopy (XPS), since they provide information on the chemical environment of the sulfur atoms in the thiophene backbones. The deconvolution of the S2p core levels of B(C_6_F_5_)_3_‐ and Mes_2_B^+^[B(C_6_F_5_)_4_]^−^‐doped P3HT is shown in **Figure** **5**a,b, respectively. The S2p spectrum of pristine P3HT shows the typical doublet with a spin–orbit splitting between the S2p_3/2_ and S2p_1/2_ components of 1.18 eV and an intensity ratio of 1:2, as expected for an S2p doublet.^[^ ^68^ ^]^ With increasing dopant concentration, the S2p levels for both types of dopants shift toward lower binding energy because the Fermi level, which is the energy reference for photoemission measurements, shifts within the bandgap of P3HT (vide infra). For B(C_6_F_5_)_3_‐doped P3HT films, the S2p spectra can be fitted with two doublets with an energy difference of 0.4 eV. The additional doublet at 0.4 eV higher binding energy, compared to undoped P3HT, increases in intensity with increasing dopant concentration and can thus be assigned to the signal of polarons (cationic segments on a polymer chain). For Mes_2_B^+^[B(C_6_F_5_)_4_]^−^‐doped P3HT, the spectra for dopant concentrations up to 10% can also be fitted adequately with two doublets. However, at and beyond 10% dopant concentration, an additional doublet at 2.0 eV higher binding energy, compared to the neutral component, is necessary to reproduce the experimental data with a fit. A deconvolution of the corresponding S2s core level spectra shows the same trend (see Figure S6 in the Supporting Information). XPS measurements reported for electrodeposited and doped P3HT show a similar development of the S2p line shape upon increasing dopant concentration. This was interpreted in terms of formation of polarons and bipolarons, leading to doublets shifted to higher binding energy by 0.7–0.8 and 2.0–2.1 eV, respectively, compared to the neutral component.^[^ ^26^, ^27^, ^69^ ^]^ These results are in reasonable agreement with our fitting parameters, considering that the reported values are based on different deconvolution procedures. An alternative origin of the observed components at higher binding energy in the S2p (and S2s) core levels could be doping‐enhanced shake‐up transitions, as was proposed for ClO_4_^−^‐doped poly(3‐methylthiophene);^[^ ^70^ ^]^ however, this can be ruled out in the present case as can be inferred from the discussion of Figure S14 in the Supporting Information. Importantly, the emergence of the high binding energy component assigned to bipolarons is once more correlated (in terms of dopant concentration) with the changes in the EPR and absorption spectroscopy data, as well as conductivity, providing further evidence for bipolaron formation on P3HT when doped with Mes_2_B^+^[B(C_6_F_5_)_4_]^−^.

Figure 5c shows the relative amount of P3HT monomers affected by the formation of polarons and bipolarons as deduced from the deconvolution of the S2p core levels of Figure 5b. This diagram was obtained using a refined three‐component fitting of the bipolaron S2p core levels, as suggested previously.^[^ ^71^, ^72^ ^]^ Following the reported simulation results for a hexamer model, a bipolaron leads to three equally intense S2p components, where the two lower energy components are nearly equal in binding energy to the neutral and polaron S2p core levels. Thus, it is assumed that the formation of bipolarons leads to contributions to the neutral and polaron component, which are equal in area to the third bipolaron component shifted by 2.0 eV from the neutral component. As displayed in Figure 5c, the relative amount of polarons increases up to around 15–20% dopant concentration, and then slightly decreases whereas the bipolaron fraction grows. In comparison, our EPR results show the absolute number of polarons formed at 2% and 35% dopant concentration is similar, and approximately half the maximum number of polarons (found for 10% dopant concentration), i.e., good quantitative agreement between EPR and XPS results persists. In addition, the calculated average number of charges per polymer monomer show also a very good quantitative agreement with the dopant concentrations estimated from the fluorine:sulfur atomic ratio (given in Table S1 in the Supporting Information).

### 2.4 Doping Mechanism

Our results provide evidence for the formation of polarons as the only charged species upon doping P3HT with B(C_6_F_5_)_3_. The strong similarities in the optical and EPR spectra recorded for P3HT doped with the salt Mes_2_B^+^[B(C_6_F_5_)_4_]^−^ observed at dopant concentrations below 10% indicate predominant formation of polarons as well. Comparison of the spin concentrations determined by EPR as a function of dopant concentration, however, reveal Mes_2_B^+^[B(C_6_F_5_)_4_]^−^ to have an approximately two times higher doping efficiency (number of created charges compared to number of dopants). The decrease in spin concentration and conductivity at higher dopant concentration for Mes_2_B^+^[B(C_6_F_5_)_4_]^−^, accompanied by the appearance of a high binding energy component in the S2p core levels, let us conclude that bipolarons are formed, and we can assign the features BP1‐BP4 in the optical absorption spectra (Figure 2) to P3HT bipolarons with high confidence.

In general, polarons in polymers extend over several monomer units and it is energetically unfavorable for two polarons to exist in close vicinity to each other.^[^ ^18^ ^]^ Consequently, there should be a maximum concentration of dopants, at which the formation of additional polarons is energetically not feasible. Theoretical studies have shown that doubly charged, moderately sized oligomers can support two polarons. In contrast, for oligomer lengths of less than seven units, the formation of a bipolaron can be energetically more favorable than two neighboring polarons.^[^ ^23^, ^63^, ^73^, ^74^ ^]^ These calculations were experimentally supported by a study on the reduction of oligofluorenes, where it was shown that side‐by‐side polarons can only exist on oligomers with a length of more than five repeat units.^[^ ^75^ ^]^ This apparent limit in oligomer length of seven units corresponds to a dopant concentration (as defined in the Experiment Section below) of around 14%, around which a threshold for bipolaron stability could be reached. Our optical, conductivity, XPS, and EPR data suggest this threshold to occur around 10% dopant concentration for Mes_2_B^+^[B(C_6_F_5_)_4_]^−^‐doped P3HT, as beyond this concentration, we see the formation of bipolarons. At present, it is not clear why the formation of bipolarons with B(C_6_F_5_)_3_ as dopant (or other molecular dopants, as discussed in the Introduction) does not happen. We speculate that this could be attributed i) to a higher effective oxidation strength of Mes_2_B^+^[B(C_6_F_5_)_4_]^−^ compared to other dopants (facilitating the creation of further holes in the already highly doped P3HT), ii) to the bulkiness of the [B(C_6_F_5_)_4_]^−^ anion and thus a different electrostatic potential landscape on the molecular length scale in films,^[^ ^76^ ^]^ and iii) to dopant‐induced changes in morphology that render bipolaron formation more favorable. We recall at this point our observations in absorption spectra, which indicated that beyond ≈5% dopant concentration aggregation of P3HT was effectively suppressed. In contrast, up to at least 50% of B(C_6_F_5_)_3_ concentration we found strong aggregation in doped P3HT (vide supra).

The doping mechanism of P3HT with boron‐based Lewis acids such as B(C_6_F_5_)_3_ was recently revealed to occur by the formation of a strongly acidic B(C_6_F_5_)_3_:H_2_O complex, protonation of parts of the polymer chain, and subsequent electron transfer from a neutral chain segment to a positively charged, protonated one.^[^ ^49^ ^]^ The mechanism is different for other widely used dopants, like F_4_TCNQ and F_6_TCNNQ, where an electron is directly transferred from the semiconductor to the p‐dopant, and the ionized dopant then acts as the counteranion. For Mes_2_B^+^[B(C_6_F_5_)_4_]^−^, the proposed doping mechanism is schematically depicted in Figure 5d. Electron transfer occurs from a segment of P3HT to the boron cation, forming the neutral radical Mes_2_B^•^. The positively charged polaron on P3HT is stabilized by [B(C_6_F_5_)_4_]^−^. For dopant concentrations above 10%, where the formation of two neighboring polarons is energetically unfavorable, bipolarons are formed with electron transfer in an analogous manner as for the polaron. Thus, one would expect that one (two) Mes_2_B^•^ radicals are present after each polaron (bipolaron) formation process. The highly reactive Mes_2_B^•^ has previously been described to form a dimesitylborane dimer (Mes_2_BH)_2_ after oxidation of decamethylferrocene followed by hydrogen abstraction.^[^ ^51^, ^77^ ^]^ A similar reaction could occur during our doping process. Our EPR data exclude the presence of a paramagnetic dopant species, such as a radical. Furthermore, XPS scans of the B1s region for Mes_2_B^+^[B(C_6_F_5_)_4_]^−^‐doped P3HT films show only the presence of negatively charged boron, which increases with increasing dopant concentration (see Figure 7 in the Supporting Information). From both methods, we thus conclude that no neutral radical nor cation of Mes_2_B is present in our samples, and also no (Mes_2_BH)_2_. Consequently, as shown in Figure 5d, we suggest that Mes_2_B^•^ and (Mes_2_BH)_2_ (if formed) are sufficiently volatile to leave during film processing, either already in solution or during film drying. The weakly coordinating counteranion [B(C_6_F_5_)_4_]^−^ has been proposed to lead to increased air‐ and moisture‐stability of doped carbon nanotubes and graphene films due to its chemical inertness, thermal stability and hydrophobic nature.^[^ ^51^, ^52^ ^]^ Finally, the fact that [B(C_6_F_5_)_4_]^−^ is a nonradical and has a comparably large size, may be beneficial for stabilizing the induced charges on the polymer chains.^[^ ^78^ ^]^

### 2.5 Valence Electronic Structure of P3HT with Polarons and Bipolarons

**Figure 6:**
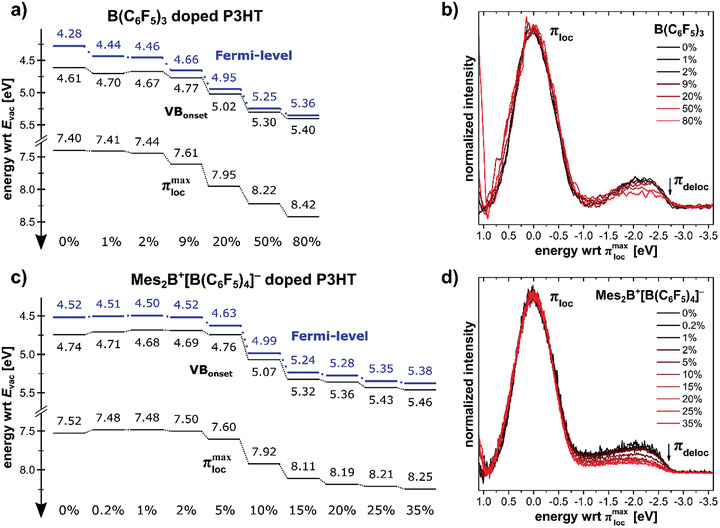
Energy level diagrams derived from UPS measurements and comparison of valence electron regions. For a) B(C_6_F_5_)_3_‐ and c) Mes_2_B^+^[B(C_6_F_5_)_4_]^−^‐doped P3HT with respect to the vacuum level (*E*_vac_) showing the shift of the Fermi level toward the valence band, followed by Fermi level pinning around 80 meV above the valence band onset (VB_onset_). (b) and (d) show the valence band spectra of B(C_6_F_5_)_3_‐ and Mes_2_B^+^[B(C_6_F_5_)_4_]^−^‐doped P3HT, respectively, after numerical Shirley‐background correction and normalization with respect to the localized *π*‐band (*π*_loc_) intensity.

Changes of the electronic properties of P3HT films upon increased dopant concentration were investigated with ultraviolet photoemission spectroscopy (UPS). **Figure** **6**a,c shows the corresponding schematic energy level diagrams for B(C_6_F_5_)_3_‐ and Mes_2_B^+^[B(C_6_F_5_)_4_]^−^‐doped P3HT (all UPS spectra are shown in Figure S8 in the Supporting Information). Upon doping, the Fermi level shifts toward the valence band (VB), and eventually becomes pinned at ≈50 meV for B(C_6_F_5_)_3_ and at ≈80 meV for Mes_2_B^+^[B(C_6_F_5_)_4_]^−^ above the valence band onset (corresponding to the valence band maximum), at dopant concentrations of ≈20% and 10%, respectively. For dopant concentrations up to ≈5%, the sample work function and the valence band onset move in parallel, keeping the ionization energy constant at ≈4.7 eV, in good agreement with previously published values.^[^ ^79^, ^80^, ^81^ ^]^ However, for dopant concentrations of 10% and higher, the work function markedly increases up to almost 5.4 eV, which, in turn, results in a likewise strong increase of ionization energy. A similar increase in ionization energy was also observed for electrodeposited and doped thin films of P3HT from UPS measurements.^[^ ^27^, ^69^ ^]^ This could, in principle, be due to an accumulation of anions at the film surface. However, it is worthwhile also analyzing in detail the impact of doping on the topmost valence band of the polymer (Figure 6b,d). The valence band spectrum of pristine P3HT exhibits a pronounced peak at around 3 eV binding energy with respect to the Fermi level, which originates from electron emission from the nonbonding localized *π*‐band (*π*_loc_) of the polymer. The topmost *π*‐band is delocalized along the polymer chain (*π*_deloc_) and highly dispersive in energy, resulting in a comparably flat and about 2 eV broad feature at the low binding energy side of *π*_loc_.^[^ ^80^, ^81^, ^82^ ^]^ Figure 6b,d shows the (Shirley‐type background corrected) valence band spectra of B(C_6_F_5_)_3_‐ and Mes_2_B^+^[B(C_6_F_5_)_4_]^−^‐doped P3HT films, respectively, normalized in intensity to the maximum of the localized *π*‐band ($\pi_{\mathrm{loc}}^{\max}$), which is set to zero energy for each spectrum to allow a better comparison of change in the topmost valence band. Remarkably, the shape of *π*_loc_ stays nearly unchanged for both dopants and all dopant concentrations, while the intensity of *π*_deloc_ is significantly reduced. For Mes_2_B^+^[B(C_6_F_5_)_4_]^−^, this decrease already sets in for nominal dopant concentration of 10%, while for B(C_6_F_5_)_3_, it can only be seen for very high nominal dopant concentration (at and above 50%). As noted above, the doping efficiency of the salt is about twice that of B(C_6_F_5_)_3_, yet the *π*_deloc_ intensity reduction is much stronger for Mes_2_B^+^[B(C_6_F_5_)_4_]^−^. While the width of *π*_deloc_ does not seem to be affected by p‐type doping, the loss in intensity can be interpreted as consequence of the substantial electron transfer to the dopants. The increase in ionization energy might thus be related to a significant increase in hole density in the delocalized *π*‐band near the Fermi level. However, future studies should focus on electrostatic effects in the bulk material, as Madelung (Coulomb) interactions with counter anions can markedly shift the energies of frontier orbitals in doped polymers.^[^ ^83^ ^]^

### 2.6 Influence of Atmosphere on Doped P3HT Films

**Figure 7:**
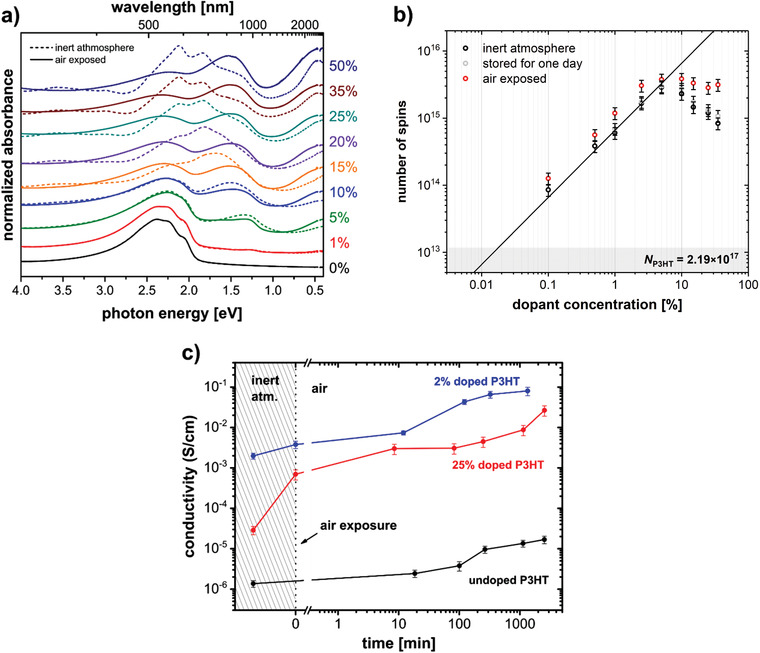
Changes of absorption, spin density, and conductivity of Mes_2_B^+^[B(C_6_F_5_)_4_]^−^‐doped P3HT upon air exposure. a) Optical absorption spectra of Mes_2_B^+^[B(C_6_F_5_)_4_]^−^‐doped P3HT before (dashed) and after (solid) air exposure. b) Number of unpaired spins *N* from EPR measurements on Mes_2_B^+^[B(C_6_F_5_)_4_]^−^‐doped P3HT before (black) and after air exposure (red). The corresponding EPR spectra are shown in Figure S9 in the Supporting Information. Before air exposure, the samples were stored for 1 day and measured again (gray) to show the stability of bipolarons when stored under inert atmosphere. c) Time‐dependent conductivity of pristine, 2%, and 25% Mes_2_B^+^[B(C_6_F_5_)_4_]^−^‐doped P3HT upon air exposure. The time axis zero indicates the start of air exposure.

From an application point of view, it is interesting to study the influence of air exposure on doped polymer semiconductors, and here particularly the stability of bipolarons. Optical absorption spectra of Mes_2_B^+^[B(C_6_F_5_)_4_]^−^‐doped P3HT films for various dopant concentrations were measured right after preparation in inert atmosphere and directly after air exposure (see **Figure** **7**a). Upon exposure to air, the absorption spectra of the doped films are significantly changed, especially for highly doped samples. Essentially all diagnostic bipolaron features vanish (most notably for BP3 and BP4), and at the same time the polaron features at around 0.4 eV (P1) and 1.5 eV (P2) increase in intensity. The transformation of EPR‐silent bipolarons to polarons is consistent with EPR measurements as shown in Figure 7b. The number of unpaired spins in the samples with dopant concentrations below 10% increases slightly, which is in line with the optical spectra and in part due to oxygen doping of P3HT (vide infra). For dopant concentrations above 10%, the increase in the number of unpaired spins upon air exposure is much larger, and saturation of the number of spins in air is reached.

The influence of bipolarons on charge transport in P3HT was investigated by additional conductivity measurements before and after air exposure for two samples with different dopant concentrations: a 2% doped sample, where the charge carriers are mainly polarons, and a 25% doped sample containing a significant amount of bipolarons (compare Figure 5c). Reference measurements on samples prepared at the same time and kept under inert atmosphere showed the conductivity of the doped samples to be fairly stable (within the same order of magnitude on that time scale). Directly after preparation, P3HT exhibits a conductivity of around 10^−6^ S cm^−1^, 2% doped P3HT of 2 × 10^−3^ S cm^−1^, and 25% doped P3HT of around 3 × 10^−5^ S cm^−1^ (differences compared to Figure 3 are attributed to the use of different batches of P3HT). Figure 7c shows the time dependent change in conductivity, where *t* = 0 min marks the moment of air exposure. Upon air exposure, the conductivity of pristine P3HT slowly increases by about one order of magnitude within several hours. This increase in conductivity is attributed to p‐doping of P3HT by oxygen, which proceeds slowly and saturation of the conductivity is reached after around 4–5 h, similar to what has been reported previously.^[^ ^84^, ^85^ ^]^ The 2% doped sample shows a similar increase in conductivity over nearly two orders of magnitude over the same time scale. This shows that also already molecularly doped samples are further p‐doped by oxygen as also observed by absorption and EPR. Note that the samples were exposed to air and not to pure oxygen, thus the influence of oxygen and/or water cannot be differentiated. Nonetheless, it is assumed at this point that the main influence is due to oxygen. For B(C_6_F_5_)_3_‐doped samples, a similar effect of further p‐doping via air is also observed (see Figure S10 in the Supporting Information). In contrast, the increase in conductivity of the 25% Mes_2_B^+^[B(C_6_F_5_)_4_]^−^‐doped sample sets in rapidly, indicating the most likely cause to be oxygen, which is expected to diffuse faster through the film than water. It increases by about one order of magnitude already directly after air exposure, and by overall two orders of magnitude within the first 10 min, nearly reaching the conductivity of the only 2% doped sample. Over the whole measurement time of 44 h, the conductivity of the air exposed 25% doped film increased by a total of three orders of magnitude, whereas the reference sample in inert atmosphere remained at its original conductivity level. In accordance with the results from optical and EPR spectroscopy, the rapid conductivity increase of the 25% doped sample is due to a transformation of bipolarons back to polarons upon air exposure, while the additional slow increase over several hours is most likely also related to doping by oxygen. The rapid increase in conductivity over two orders of magnitude associated with the bipolaron to polaron transition also indicates that bipolaronic charge carriers are less mobile than polaronic charge carriers, assuming that the total number of holes is maintained during the transition. Such a behavior was also observed for the doping of P3HT with FeCl_3_, where the mobility of polarons was estimated to be two orders of magnitude higher than that of bipolarons.^[^ ^28^ ^]^ This difference in mobility can most likely be related to i) bipolarons inducing stronger polarization in their vicinity and consequently carrying a larger polarization cloud, which effectively decreases mobility, and ii) an increased Coulomb localization near counterions, effectively increasing the activation energy for carrier release. However, from these experiments we cannot infer the mechanism by which bipolarons are reverted to polarons.

### 2.7 Doping of Further Polymers and Effective Electron Affinity

Electron transfer from an already charged (polaronic) P3HT chain segment to the Mes_2_B^+^ cation is energetically possible (see Section 2.3). The IE of P3HT polarons can be estimated from the valence region spectra (see Figure 6 and Figure S8 in the Supporting Information). While the film of neutral P3HT has an IE of about 4.7 eV, the IE for a film containing mostly P3HT polarons is about 5.4 eV, i.e., P3HT films doped with a high concentration of B(C_6_F_5_)_3_. This IE difference is larger, but yet within the same order of magnitude as the difference between the first and second oxidation process (several 100 mV) measured for P3HT by cyclic voltammetry.^[^ ^29^, ^86^ ^]^ Therefore, the effective EA of Mes_2_B^+^[B(C_6_F_5_)_4_]^−^ should be equal to or higher than 5.4 eV, so that electron transfer from polaronic P3HT to Mes2B^+^ is possible, to give rise to the bipolaron stabilized by [B(C_6_F_5_)_4_]^−^. This estimate is in line with a calculated EA value for Mes_2_B^+^ of 5.4 eV.^[^ ^43^ ^]^ However, this calculation was done for an isolated molecule in the gas phase, so that the actual EA in the solid might be even higher due to polarization.

**Figure 8:**
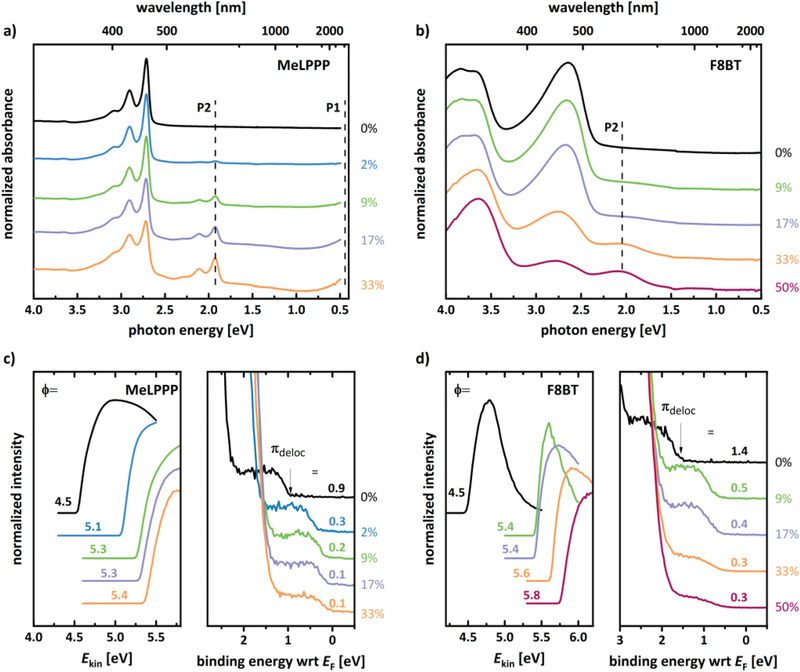
Optical absorption and photoemission spectroscopy data showing p‐type doping of MeLPPP and F8BT by Mes_2_B^+^[B(C_6_F_5_)_4_]^−^. Absorption spectra for a) MeLPPP and b) F8BT films doped with Mes_2_B^+^[B(C_6_F_5_)_4_]^−^. For MeLPPP, the typical optical transitions P1 and P2 at 0.4 and 1.9 eV, respectively, are observed and marked with dotted lines. For F8BT, only the polaron feature P2 at 2.0 eV can be seen, while the P1 NIR band is outside the spectral range. All spectra are normalized to their highest absorbance peak and vertically shifted for clarity. UPS SECO (left) and valence band (right) spectra of c) MeLPPP and d) F8BT films doped with Mes_2_B^+^[B(C_6_F_5_)_4_]^−^. Φ is the work function, and the numbers next to valence spectra give the valence band onset binding energy with respect to the Fermi level *E*_F_.

To test the doping strength of Mes_2_B^+^[B(C_6_F_5_)_4_]^−^ further, we investigated mixtures of the organic salt with materials of IE values higher than P3HT, i.e., the polymers MeLPPP (IE ≈ 5.4 eV) and F8BT (IE ≈ 5.9 eV) as well as the fullerene C_60_ (IE > 6.1 eV).^[^ ^87^, ^88^ ^]^ The optical absorption spectra of Mes_2_B^+^[B(C_6_F_5_)_4_]^−^‐doped MeLPPP and F8BT films are shown in **Figure** **8**. The absorption spectrum of an undoped MeLPPP film in Figure 8a shows the typical absorption features at 2.7, 2.9, and 3.1 eV, the latter two being vibronic progressions of the 0‐0 transition, and well resolved due to the planarized conjugated backbone.^[^ ^89^ ^]^ The doped MeLPPP spectra show the emergence of P1 (at least the onset of this peak) and P2 (at 1.9 eV) transitions of positive MeLPPP polarons, which were reported to lie at 0.4 and 1.9 eV, respectively. ^[^ ^89^, ^90^ ^]^ These values are in good agreement with our observations. Both features increase in intensity with increasing dopant concentration, indicating a higher amount of polarons. Simultaneously, the *π*–*π* ^*^ band at 2.7 eV bleaches as the amount of neutral MeLPPP segments is reduced. Figure 8b shows the absorption spectrum of undoped and Mes_2_B^+^[B(C_6_F_5_)_4_]^−^‐doped F8BT films. The absorption features of the neutral polymer around 2.7, 3.7, and 3.8 eV are also in agreement with literature.^[^ ^91^ ^]^ Due to the limited energy range of our setup, the P1 NIR band cannot be observed for the doped polymer. Nevertheless, the transition P2 peaking just below 2.0 eV provides clear evidence for doping‐induced polaron formation.^[^ ^91^ ^]^ The polaron feature increases in intensity with increasing dopant concentration and the *π*–*π* ^*^ band at 2.7 eV bleaches accordingly. The spectra of Mes_2_B^+^[B(C_6_F_5_)_4_]^−^‐doped polymer films did not show any significant changes upon air exposure for MeLPPP and only a small reduction of the polaron signature for F8BT (spectra shown in Figure S11 in the Supporting Information), suggesting polarons being fairly stable in both polymers.

For comparison, we attempted doping MeLPPP and F8BT with the strong p‐dopant F_6_TCNNQ, the EA of which is around 5.6 eV.^[^ ^49^ ^]^ We recall that doping P3HT with F_6_TCNNQ resulted in polarons only, no indications for bipolaron formation was found.^[^ ^33^ ^]^ The absorption spectra of MeLPPP and F8BT solutions mixed with F_6_TNNCQ are shown in Figure S12 in the Supporting Information, but no absorption features diagnostic of polaron formation can be observed. Since Mes_2_B^+^[B(C_6_F_5_)_4_]^−^ is able to induce polarons in both polymers, but F_6_TCNNQ is not, it is thus reasonable to conclude that the effective EA of the salt is higher than that of F_6_TCNNQ (5.6 eV). Ultimately, we mixed Mes_2_B^+^[B(C_6_F_5_)_4_]^−^ and C_60_ in solution (spectra shown in Figure S12 in the Supporting Information). No indications for doping of C_60_ by the salt were observed, implying that the effective EA of Mes_2_B^+^[B(C_6_F_5_)_4_]^−^ is lower that the IE of C_60_ (>6.1 eV).

To further investigate the p‐doping capabilities of Mes_2_B^+^[B(C_6_F_5_)_4_]^−^, the conductivity of MeLPPP upon doping was investigated (see Figure S13 in the Supporting Information). An up to almost six orders of magnitude increase in conductivity was observed, up to 4 × 10^−4^ S cm^−1^, for dopant concentrations up to around 9%. Noteworthily, the conductivity of Mes_2_B^+^[B(C_6_F_5_)_4_]^−^‐doped MeLPPP films decreased by less than one order of magnitude after 1 month air exposure. For pristine and Mes_2_B^+^[B(C_6_F_5_)_4_]^−^‐doped F8BT films, the conductance of all samples was too low to provide a reliably signal in our setup. Although absorption measurements strongly suggest high abundance of doping‐induced polarons in F8BT (see Figure 8b), the conductivity remained below ≈10^−9^ S cm^−1^. This behavior might be due to reasons suggested in literature, i.e., holes might localize on donor moieties of the donor–acceptor polymer, leading to more localized polarons and thus lower conductivity.^[^ ^91^ ^]^ Despite the low conductivity, photoemission measurements—as discussed in the following—do provide further evidence for p‐doping of F8BT with Mes_2_B^+^[B(C_6_F_5_)_4_]^−^.

Changes of the electronic properties of MeLPPP and F8BT films due to doping with Mes_2_B^+^[B(C_6_F_5_)_4_]^−^ were investigated with UPS (see Figure 8c,d). For both polymers we observe a strong increase in sample work function upon increased dopant concentration, i.e., by up to 0.9 eV for MeLPPP and 1.3 eV for F8BT. Also, for both polymers, the onset of the delocalized topmost valence band (*π*_deloc_) shifts toward the Fermi level, i.e., by up to 0.8 eV for MeLPPP and 1.1 eV for F8BT, essentially in parallel with the work function shift. Thus, the IE of MeLPPP slightly increases by 0.1 to 5.5 eV, and that of F8BT increases from 5.9 eV (undoped) to 6.1 eV (50% dopant concentration). The valence band onset is only 0.1 eV below the Fermi level for MeLPPP and 0.3 eV for F8BT, which is a clear manifestation of strong p‐type doping of both polymers with Mes_2_B^+^[B(C_6_F_5_)_4_]^−^ . Since the IE of undoped F8BT is 5.9 eV and electron transfer to the dopant is possible, we estimate the effective electron affinity of Mes_2_B^+^[B(C_6_F_5_)_4_]^−^ also to be around 5.9 eV. This places this salt at the top of presently available molecular p‐type dopants, approximately at par only with the small molecule CN6‐CP,^[^ ^92^ ^]^ for which bipolaron formation in P3HT was not yet demonstrated.

## 3 Conclusion

In summary, we introduced the organic salt Mes_2_B^+^[B(C_6_F_5_)_4_]^−^ as novel p‐type dopant for polymer semiconductors, with apparent superior doping capability. With a range of complementary experimental methods, the doping mechanism was unraveled to consist of electron transfer from the polymer to Mes_2_B^+^, and stabilization of the positive charge on a polymer segment by [B(C_6_F_5_)_4_]^−^, as summarized in Figure 5d. Importantly, the former salt cations leave during film fabrication, possibly also in the form of dimers. Therefore, no adverse side products are left in thin films, which could impede charge transport or stability via subsequent reactions. The conductivity of doped P3HT films is not negatively affected by annealing up to 100 °C. In addition to the formation of polarons in the prototypical P3HT, the novel dopant also facilitates the formation of bipolarons with a high proportion beyond ≈10% dopant concentration. No other molecular dopant was yet reported with such a capability. Mes_2_B^+^[B(C_6_F_5_)_4_]^−^ was also an effective p‐type dopant for polymers with much higher ionization energy than P3HT, as shown here for MeLPPP (IE ≈ 5.4 eV) and F8BT (IE ≈ 5.9 eV). Since electron transfer to C_60_ (IE > 6.1 eV) did not proceed, we estimate the effective electron affinity of Mes_2_B^+^[B(C_6_F_5_)_4_]^−^ close to 5.9 eV. This is similar to that of the molecular dopant CN6‐CP, but the about three times higher molecular weight of [B(C_6_F_5_)_4_]^−^ and its bulkiness may be beneficial for thermal stability of films. The dopant salt also outperforms other widely used dopants, such as B(C_6_F_5_)_3_ and F_6_TCNNQ, in terms of doping efficiency and the ability to p‐dope high ionization energy organic semiconductors. Our study also opens up new research questions to be addressed in the future. These include the mechanism that transforms bipolarons in P3HT into polarons upon exposure to air, the reason for the persistent low conductivity of Mes_2_B^+^[B(C_6_F_5_)_4_]^−^‐doped F8BT despite the apparent polaron formation and Fermi level shift, the role of salt cations other than Mes_2_B^+^, and improved routes toward improved film processing to achieve even higher conductivity. Nonetheless, with this organic salt as p‐dopant, the parameter space for fundamental and applied research is now substantially extended.

## 4 Experimental Section

##### Sample Preparation

Poly(3‐hexylthiophene‐2,5‐diyl) (P3HT; weight average molecular weight *M*_w_ of 50–100 kg mol^−1^, regioregularity >90%, Sigma‐Aldrich GmbH) was used for doping studies with Mes_2_B^+^[B(C_6_F_5_)_4_]^−^, and another P3HT batch (*M*_w_ = 60.2 kg mol^−1^, regioregularity of 97.6%, Merck KGaA) for the doping studies with B(C_6_F_5_)_3_. The MeLPPP with a weight average molecular weight of 82 kg mol^−1^ was synthesized as described elsewhere.^[^ ^93^ ^]^ Poly[(9,9‐dioctylfluorenyl‐2,7‐diyl)‐*co*‐(1,4‐benzo‐{2,1′,3]thiadiazole)] (F8BT; *M*_w_ of 10 kg mol^−1^) was purchased from H.W. Sands Corp., C_60_ from Sigma‐Aldrich GmbH, tris(pentafluorophenyl)borane [B(C_6_F_5_)_3_] from TCI Deutschland GmbH, and hexafluoro‐tetracyano‐naphthoquinodimethane (F_6_TCNNQ) from Novaled GmbH. The organic salt consisting of a diarylborinium cation [Mes_2_B^+^, Mes (mesityl) = 2,4,6‐trimethylphenyl] and a tetrakis(pentafluorophenyl)borate anion ([B(C_6_F_5_)_4_]^−^) was synthesized as previously described.^[^ ^42^, ^43^ ^]^ All materials were used without further purification. Chlorobenzene (CB) and 1,2‐dichlorobenzene (*o*‐DCB) were purchased as anhydrous solvents from Sigma‐Aldrich GmbH (>99.9% purity, inhibitor‐free) and further degassed via three freeze–pump–thaw cycles. Stock solutions with concentrations of 5–50 mg mL^−1^ were prepared under nitrogen atmosphere in a glovebox (<0.1 ppm H_2_O, <0.1 ppm O_2_) using dried and degassed solvents. The stock solutions were stirred overnight to enable complete dissolution of the materials and were subsequently used within a few days. For Mes_2_B^+^[B(C_6_F_5_)_4_]^−^, *o*‐DCB was used as solvent and the mixed solutions were always prepared from the stock solutions directly before deposition of thin films. For B(C_6_F_5_)_3_, CB was used as solvent and mixed solutions were stirred overnight before thin film deposition following a previously reported protocol.^[^ ^46^ ^]^ Thin films were prepared via spin‐coating using standard laboratory spin‐coaters at various speeds (1000–6000 rpm) and times (1–2 min) to achieve the desired thickness. The reported dopant concentration *c*, given in %, of the doped semiconductor polymer films was based on the number of dopant molecules *N*_D_ with respect to the number of monomeric units *N*_P_ of the polymers, in the form *c*  = *N*_D_/(*N*_D_ + *N*_P_). P3HT films are often thermally annealed in order to increase the crystallinity of the thin films and thus conductivity.^[^ ^81^ ^]^ However, it was found that the usual annealing of doped P3HT films leads to a difficult to control loss of dopants and was thus not done for the samples discussed here.

##### Optical Absorption Spectroscopy

Optical absorption spectroscopy was performed using a Lambda 950 UV/vis/NIR spectrophotometer (Perkin Elmer Inc.). Doped semiconductor polymer films were prepared on quartz glass substrates with thicknesses between 20 and 100 nm. The optical measurements were performed with the samples mounted in small nitrogen filled boxes with two quartz glass windows, sealed using a vinyl gasket. A baseline spectrum of the box with a mounted clean quartz glass substrate was subtracted from the spectra before further analysis.

##### Thin Film Conductivity Measurements

Thin film conductivity measurements were performed under inert atmosphere using prepatterned glass substrates with an interdigitated indium tin oxide (ITO) electrode structure. The prepatterned substrates, consisting of 5 individual devices, each comprising 100 nm thick electrodes containing 5 channels of 50 µm length and 6 mm width, were purchased from Ossila Ltd. Current–voltage (*IV*) measurements were conducted using an IV test board from Ossila and a Keithley SourceMeter 2400. Given the length of the channels of 50 µm, fields did not exceed 4000 V cm^−1^, which allowed a determination of the conductivity from the Ohmic region of the *IV* characteristics. For the doped MeLPPP/F8BT samples, the transfer length method (TLM) was used to ensure that the results are not dominated by the contact resistance. For this purpose, substrates containing 5 channels with different channel lengths (50–200 µm) were used. The contact resistance was determined by the intersection of the linearly fitted resistance‐channel length (RL) curve with the *y*‐axis. The contact resistance was found to be approaching 0 Ω, thus the increase of conductivity with dopant concentration reported below was due to doping and not due to a decrease in contact resistance.

##### Scanning Force Microscopy

After completion of the *IV* measurements, film thickness was determined on samples scratched with a needle (syringe cannula) by recording scanning force microscopy height images using a Bruker Dimension Icon. Images were recorded in ScanAsyst (tapping) mode using silicon cantilevers with a typical resonant frequency of 70 kHz and a spring constant of 0.4 N m^−1^.

##### EPR Measurements

Samples for EPR measurements were prepared by filling 50 µL of doped P3HT solutions in *o*‐DCB into 3 mm inner diameter quartz tubes inside a nitrogen atmosphere glovebox, and then evaporating the solvent under vacuum, resulting in a solid film on the inner walls of the EPR tube. The EPR tubes were then backfilled with helium and flame‐sealed. All samples were prepared at a constant P3HT concentration, while varying the dopant concentration. X‐band continuous wave EPR measurements were performed using an ER 4122 SHQE resonator on a home‐built spectrometer consisting of a Bruker ER 041 MR microwave bridge with an ER 048 R microwave controller, an AEG electromagnet with a Bruker BH15 Hall effect field controller, and using a Stanford Research SR810 lock‐in amplifier with a Wangine WPA‐120 modulation amplifier for field modulation and lock‐in detection. The spectra were acquired at a microwave frequency of 9.389 GHz and a microwave power of 63 µW with a 100 kHz modulation frequency and 0.02 mT modulation amplitude. A background correction was performed with the spectrum recorded for an empty EPR tube inside the resonator cavity. The *Q*‐value of the resonator was determined from the mode picture for each measurement and used for quantitative analysis. The spectrometer was calibrated for spin quantitation with a reference sample of TEMPO in toluene with a known concentration. Three different sample series were prepared for each dopant and used to estimate the average spin concentration and the standard deviation at each dopant concentration. Additional W‐band continuous wave EPR measurements and X‐band pulse EPR measurements were also performed.

##### UPS and XPS

UPS and XPS measurements of B(C_6_F_5_)_3_‐doped P3HT as well as Mes_2_B^+^[B(C_6_F_5_)_4_]^−^‐doped MeLPPP/F8BT thin films on ITO/glass substrates were performed in a system consisting of an analysis chamber (base pressure: 10^−9^ mbar) connected to a preparation chamber (base pressure: 5 × 10^−8^ mbar). UPS was performed using a nonmonochromated helium‐gas‐discharge lamp (21.22 eV) with low photon flux (attenuated by an 800 nm aluminum filter) in order to avoid radiation damage of the samples. The excitation source for XPS was nonmonochromated Al K*α* (1486.7 eV). The spectra were collected in normal emission using a SPECS Phoibos 100 hemispherical electron energy analyzer using a pass energy of 5 eV for UPS and 50 eV (20 eV) for XPS survey (detail) scans. For Mes_2_B^+^[B(C_6_F_5_)_4_]^−^‐doped P3HT thin films on ITO/glass substrates, monochromated UPS and XPS measurements were performed in a customized SPECS UHV system, including an analysis chamber (base pressure: 2 × 10^−10^ mbar) and a preparation chamber (base pressure: 2 × 10^−10^ mbar). XPS was measured using a monochromatized Al K*α* source (1486.7 eV) and UPS using the monochromatized He I*α* excitation line (21.22 eV). The spectra were collected with a Specs PHOIBOS 150 hemispherical analyzer using a pass energy of 4 eV for UPS and 50 eV (20 eV) for XPS survey (detail) scans. All spectra were recorded at room temperature. The secondary electron cutoff (SECO) spectra were measured with a bias of −10 V applied to the sample to clear the analyzer work function. The onset of the VB and the SECO was determined by the intersection of a horizontal baseline resembling a constant background and a line fitted to the linear part of the VB or SECO, respectively. A Shirley‐type background was removed from the measured VB region spectra using a noniterative Shirley method.^[^ ^94^ ^]^

##### Statistical Analysis

Statistical analysis was not applied to the data presented in this work.

## Conflict of Interest

The authors declare no conflict of interest.

## Supporting information

Supporting Information
