# Supplementary material for: An Organic Borate Salt with Superior p‐Doping Capability for Organic Semiconductors
Source: Adv Sci (Weinh). 2020 Jul 6;7(17):2001322. doi: 10.1002/advs.202001322 (PMC7507313; doi:10.1002/advs.202001322)
Supplement: Supplementary file 1 — Supporting Information [file ADVS-7-2001322-s001.pdf]

# Supporting Information:

# An organic borate salt with superior *p*-doping capability for organic semiconductors

B. Wegner,[1,2] D. Lungwitz,[1] A. E. Mansour,[1] C. E. Tait,[3] N. Tanaka,[4] T. Zhai,[5] S. Duhm,[5] M. Forster,[6] J. Behrends,[3] Y. Shoji,[4] A. Opitz,[1] U. Scherf,[6] E. J. W. List-Kratochvil,[1,7] T. Fukushima,[4] and N. Koch[1,2,5]*

[1] *Institut für Physik & IRIS Adlershof, Humboldt-Universität zu Berlin, D-12489 Berlin, Germany*
[2] *Helmholtz-Zentrum Berlin für Materialien und Energie GmbH, D-12489 Berlin, Germany*
[3] *Berlin Joint EPR Lab, Fachbereich Physik, Freie Universität Berlin, D-14195 Berlin, Germany*
[4] *Laboratory for Chemistry and Life Science, Institute of Innovative Research, Tokyo Institute of Technology, Yokohama 226-8503, Japan*
[5] *Institute of Functional Nano & Soft Materials (FUNSOM), Jiangsu Key Laboratory for Carbon-Based Functional Materials & Devices and Joint International Research Laboratory of Carbon-Based Functional Materials and Devices, Soochow University, Suzhou 215123, P.R. China*
[6] *Makromolekulare Chemie & Institut für Polymertechnologie, Bergische Universität Wuppertal, D-42097 Wuppertal, Germany*
[7] *Institut für Chemie, Humboldt-Universität zu Berlin, D-12489 Berlin, Germany*

* norbert.koch@physik.hu-berlin.de

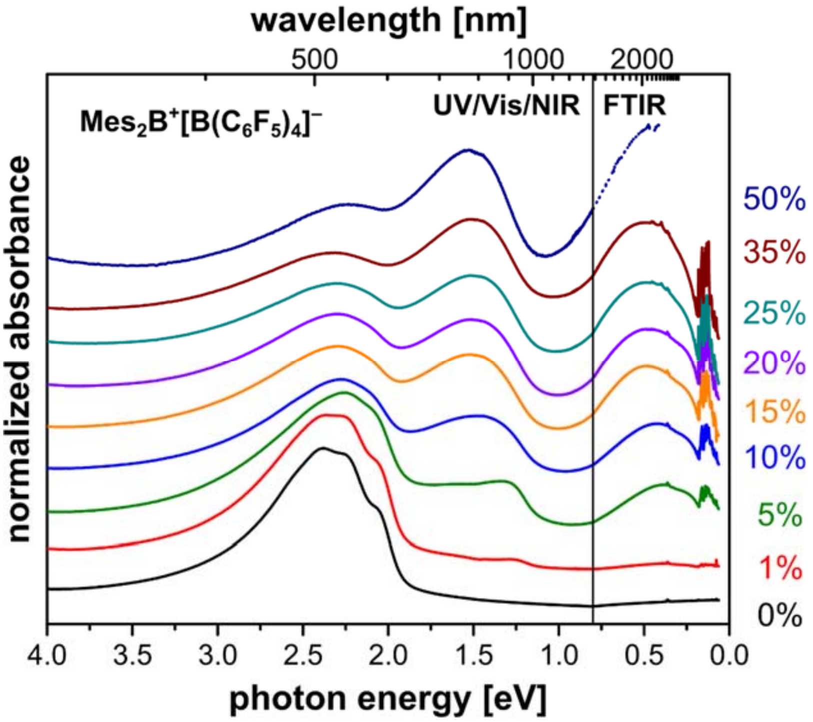


**Figure S1.** Combined optical absorption and FTIR spectra of $Mes_2B^+[B(C_6F_5)_4]^-$ doped P3HT films after air-exposure. The FTIR spectrum of the 50% doped film is missing due to poor signal-to-noise ratio, instead the NIR spectrum measured with optical spectroscopy is shown (dotted line). The structures in the region below 0.2 eV is caused by IR-active vibrations of P3HT.

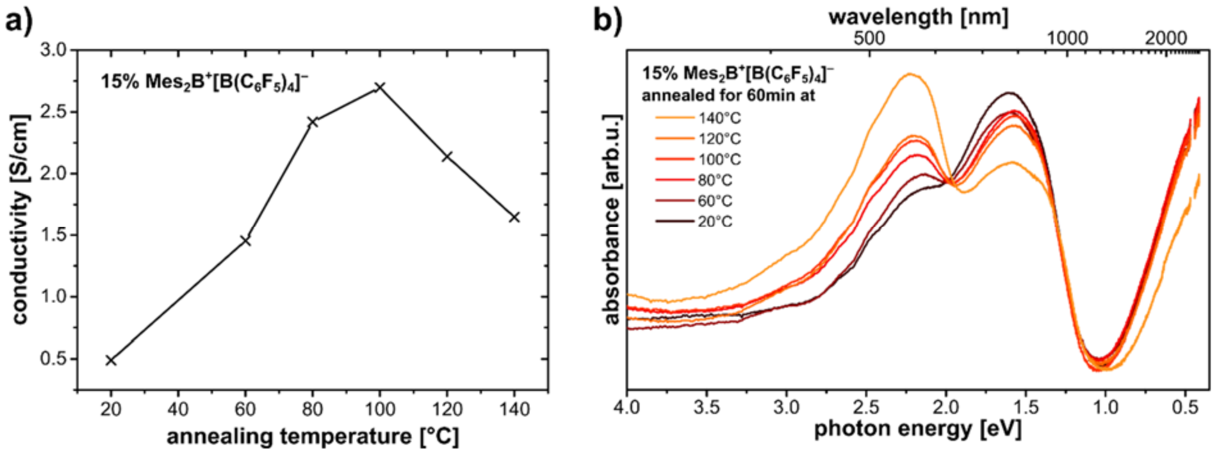


**Figure S2.** Conductivity (a) and optical absorption spectra (b) of a 15% $Mes_2B^+[B(C_6F_5)_4]^-$ doped P3HT film as a function of sample annealing temperature.

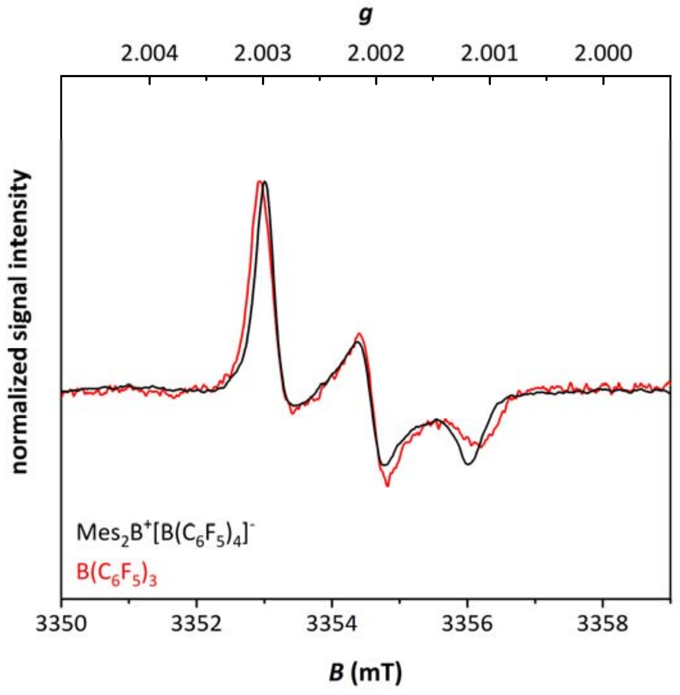


**Figure S3.** W-band continuous wave EPR spectra recorded for $B(C_6F_5)_3$ and $Mes_2B^+[B(C_6F_5)_4]^-$ doped P3HT films (dopant concentration 1%) deposited on the inside of 0.7 mm ID EPR tubes by solvent evaporation under vacuum. The measurements were performed at room temperature with a Bruker Elexsys 680 spectrometer with a Teraflex EN600-1021H resonator ($\nu_{mw}$ = 94 GHz, modulation amplitude = 0.15 mT, microwave attenuation = 27 dB, field calibration with N@C60 *g*-standard). The spectra for both dopants are almost identical and can be attributed to a single paramagnetic species, identified as the radical cation on the P3HT polymer. The principal *g*-values determined from the spectrum ($g_x$ = 2.0030, $g_y$ = 2.0020 and $g_z$ = 2.0011) are in excellent agreement with literature data for $I_2$-doped P3HT.[1] The observed W-band spectrum deviates from a powder spectrum typically expected for disordered solids and indicates preferential ordering of P3HT in the cylindrical films, created by solvent evaporation from the EPR tube, with the short in-plane axis (assigned to $g_x$ in ref. [2]) lying perpendicular to the tube walls. The substructure observed in the X-band cwEPR spectra at intermediate dopant concentrations can be attributed to the same effect.

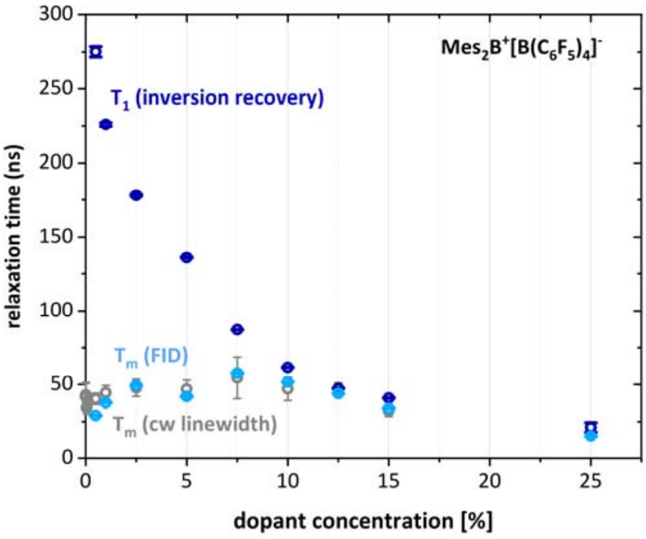


**Figure S4.** Room temperature spin lattice relaxation time $T_1$ and spin-spin relaxation time $T_m$ as a function of dopant concentration for $Mes_2B^+[B(C_6F_5)_4]^-$ doped P3HT films. The spin lattice relaxation times were determined from monoexponential fits to FID-detected inversion recovery data. The spin-spin relaxation times were determined by exponential fits of the recorded FID signals (light blue) and estimated from the linewidth of cw-EPR data (grey). The pulse EPR measurements were performed on a Bruker Elexsys 580 spectrometer with a EN 4118X-MD4 resonator. The FID was measured with a 4-step phase cycle and background corrected using an off-resonant transient. These pulse EPR measurements of the spin-lattice and spin-spin relaxation times reveal that at dopant concentrations exceeding 10%, $T_1$ is equal to $T_2$ and the corresponding cwEPR spectra are lifetime broadened. The $T_2$ values determined from the peak-to-peak EPR linewidth based on the equation $\Delta B_{pp} = \frac{2\,\hbar}{\sqrt{3} g \mu_B T_2}$ are in excellent agreement with the pulse EPR measurements.

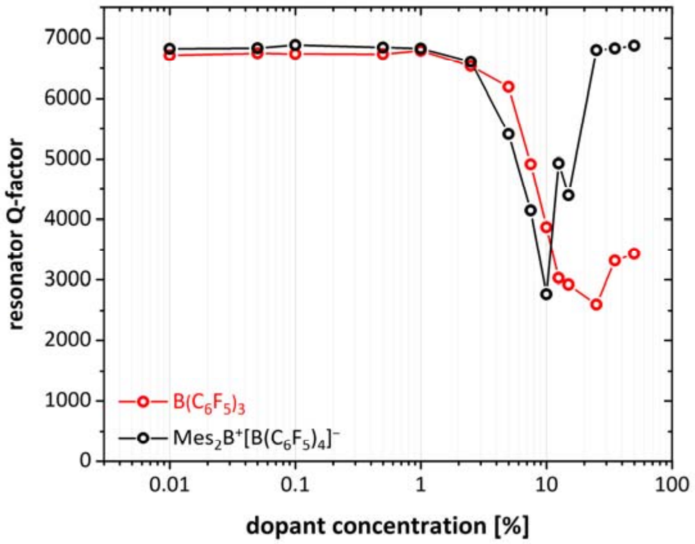


**Figure S5.** EPR resonator Q-factors determined from the mode picture for P3HT doped with $B(C_6F_5)_3$ or $Mes_2B^+[B(C_6F_5)_4]^-$ as a function of dopant concentration. The change in Q-factor provides a qualitative indication of changes in sample conductivity.

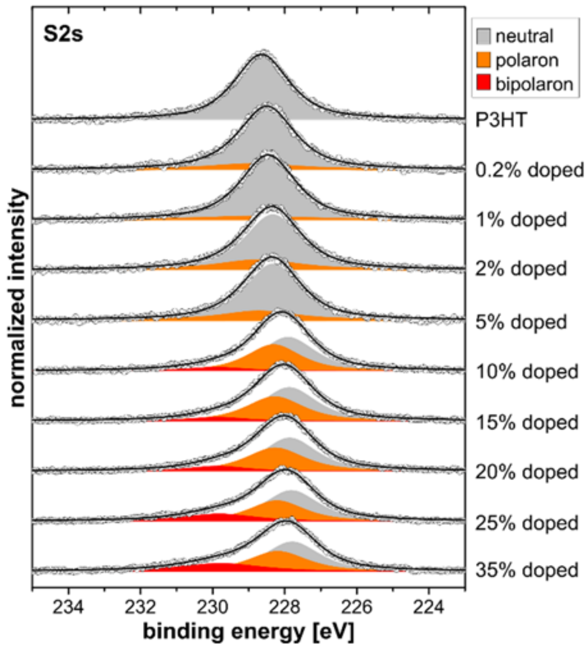


**Figure S6.** Deconvolution of the S2s core levels of $Mes_2B^+[B(C_6F_5)_4]^-$ doped P3HT in dependence of the dopant concentration. Circles display the raw data. The deconvolution of the spectra of the S2s core level is consistent with the deconvolution of the S2p core level as shown in Figure 5 of the main manuscript. The parameters for S2p and S2s were kept constant in the deconvolution. Upon doping, the core levels shift to lower binding energy, with the relative shift being very similar between S2p and S2s, while the line shape shows the emergence of new components. For the S2s core level, the areas of the polaron component at 0.4 eV and of the bipolaron component at 2.0 eV from the neutral component are very similar in their relative intensities as the S2p component areas. This shows the consistency of the used fitting procedure.

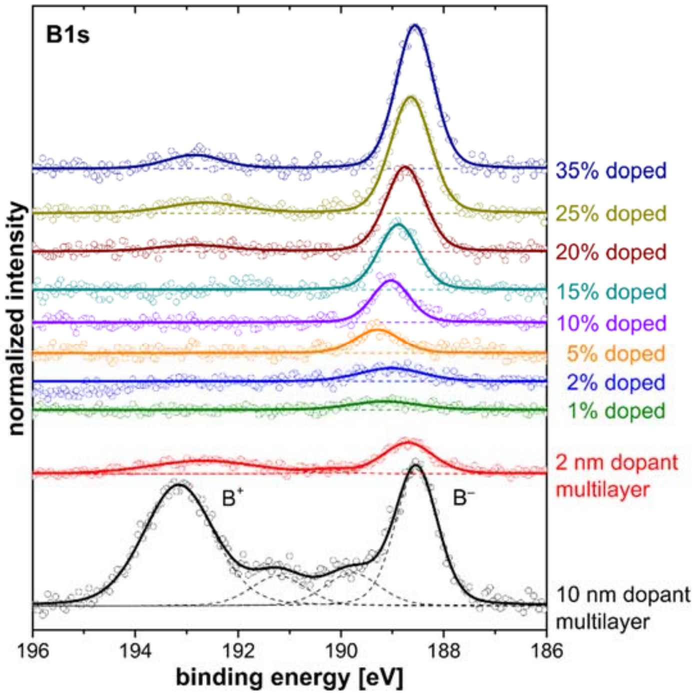


**Figure S7.** Mono-XPS detail scans of the B1s region of a 10 nm thick (black line) and a 2 nm thin (red line) multilayer of $Mes_2B^+[B(C_6F_5)_4]^-$ as well as doped P3HT films with increasing dopant concentration. The B1s spectra of the doped P3HT films are normalized with respect to the area of S2p reflecting the amount of P3HT. The spectra of the multilayers are normalized according to their thickness. The spectrum of the 10 nm thick multilayer (black line) shows two clearly distinguishable features at 193.6 eV and 188.5 eV (the small features observed in between are probably due to impurities or side-products containing neutral boron). A positive charge on an atom will yield a positive potential, which is experienced by the electron leaving the 1s core level and will therefore reduce the kinetic energy of the leaving electron. Similarly, a negative charged atom will show a higher kinetic energy and thus lower binding energy of the 1s core level. The reported binding energy of neutral boron is 189.4 eV, which lies between the two observed features.[3] The feature at higher binding energy can thus be ascribed to the positively charged boron in $Mes_2B^+$ and the feature at 188.5 eV is assigned to the negatively charged boron in $[B(C_6F_5)_4]^-$. The spectrum of the 2 nm thin multilayer also shows two main features, which are separated by 4 eV from each other. In contrast to the thick multilayer, where both features have nearly the same intensity, here, the feature of $B^+$ shows only ca. half the intensity of the one of $B^-$. This can be related to $Mes_2B^+$ also undergoing a reaction with the ITO substrate used in the PES measurements. The electrons are proposed to be transferred from the ITO to $Mes_2B^+$, which leads to the formation of an interface dipole at the ITO interface. This was found to increase the effective work function from 4.6 eV for bare ITO to around 5.8 eV for both multilayers. For the thin multilayer, the B1s spectrum, however, shows no feature at equal intensity which could be attributed to neutral boron formed after reduction of $Mes_2B^+$. The lack of such a feature is even more striking in the case of the doped P3HT films also shown in this figure. Here, only the feature of $B^-$ is observed, the intensity of which is increasing with increasing dopant concentrations (the shift in the core level can be associated with the shift in Fermi level). For dopant concentrations above 20%, a small intensity feature of $B^+$ can be observed, which is most likely due to a small portion of non-reacted ionic salt.

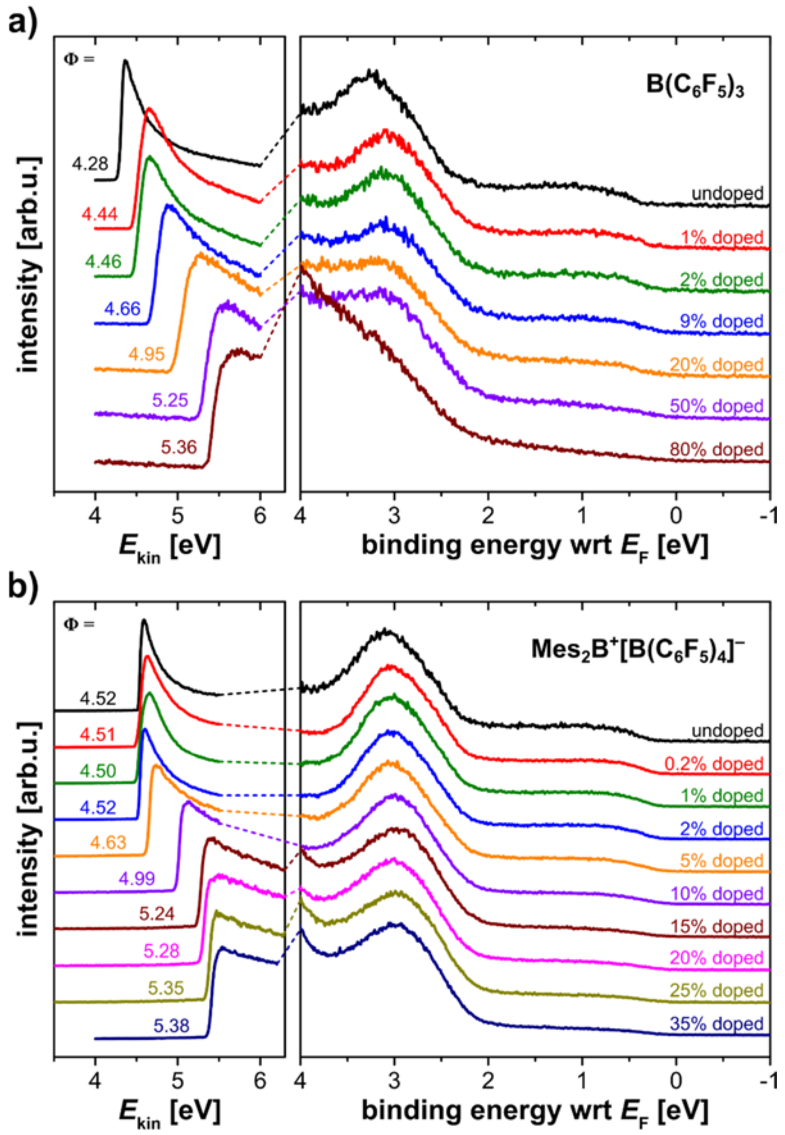


**Figure S8.** (Mono-)UPS spectra of **a)** $B(C_6F_5)_3$ and **b)** $Mes_2B^+[B(C_6F_5)_4]^-$ doped P3HT films. The left panel displays the secondary electron cutoff (SECO) spectra with the work function values (ϕ), while the right panel shows the valence band region. The differences in work function between undoped P3HT in a) and b) are due to different ITO substrates (different batches of the same supplier), which, however, were the same within one experimental series.

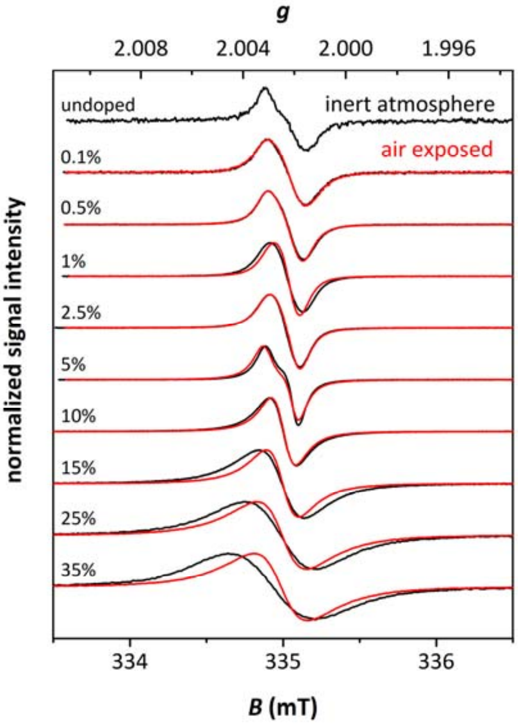


**Figure S9.** Comparison of X-band continuous wave EPR spectra recorded for $Mes_2B^+[B(C_6F_5)_4]^-$ doped P3HT films prepared in an inert atmosphere and exposed to air before the EPR measurement ($\nu_{mw}$ = 9.389 GHz).

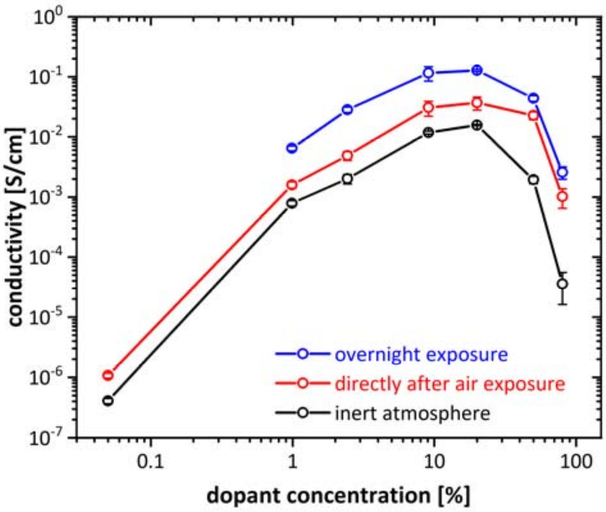


**Figure S10.** Conductivity measurements of $B(C_6F_5)_3$ doped P3HT in inert atmosphere (black), directly after air exposure (red), and after air exposure overnight (blue).

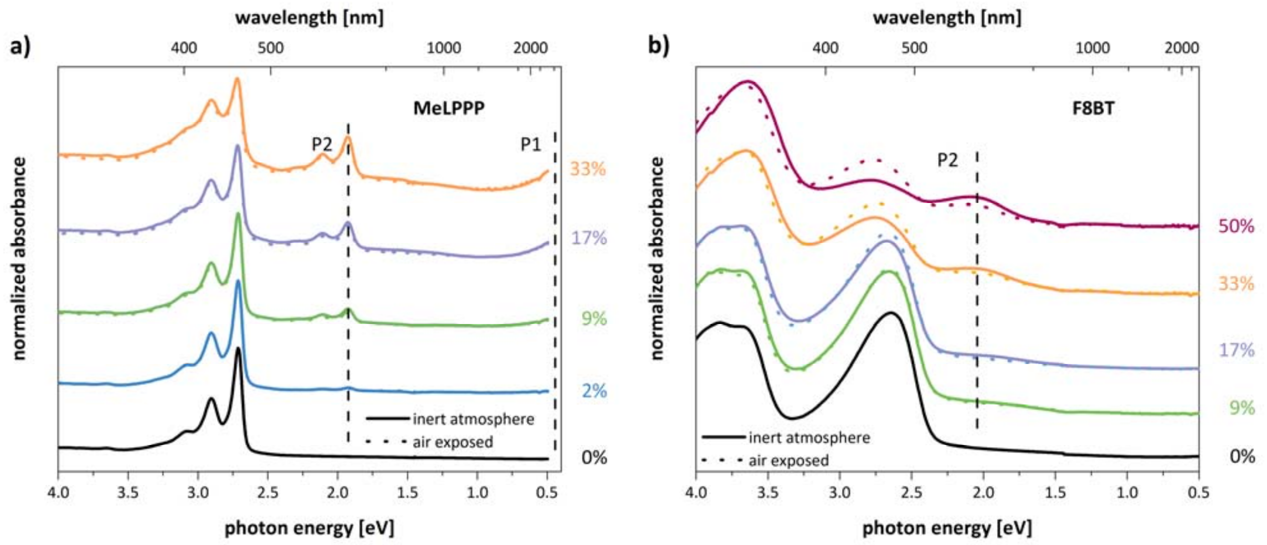


**Figure S11.** Optical absorption spectra of $Mes_2B^+[B(C_6F_5)_4]^-$ doped **a)** MeLPPP and **b)** F8BT films before (solid line) and directly after air exposure (dotted line). The optical spectra show no significant change before and after air exposure. All spectra are normalized to their highest absorbance peak and vertically shifted for clarity.

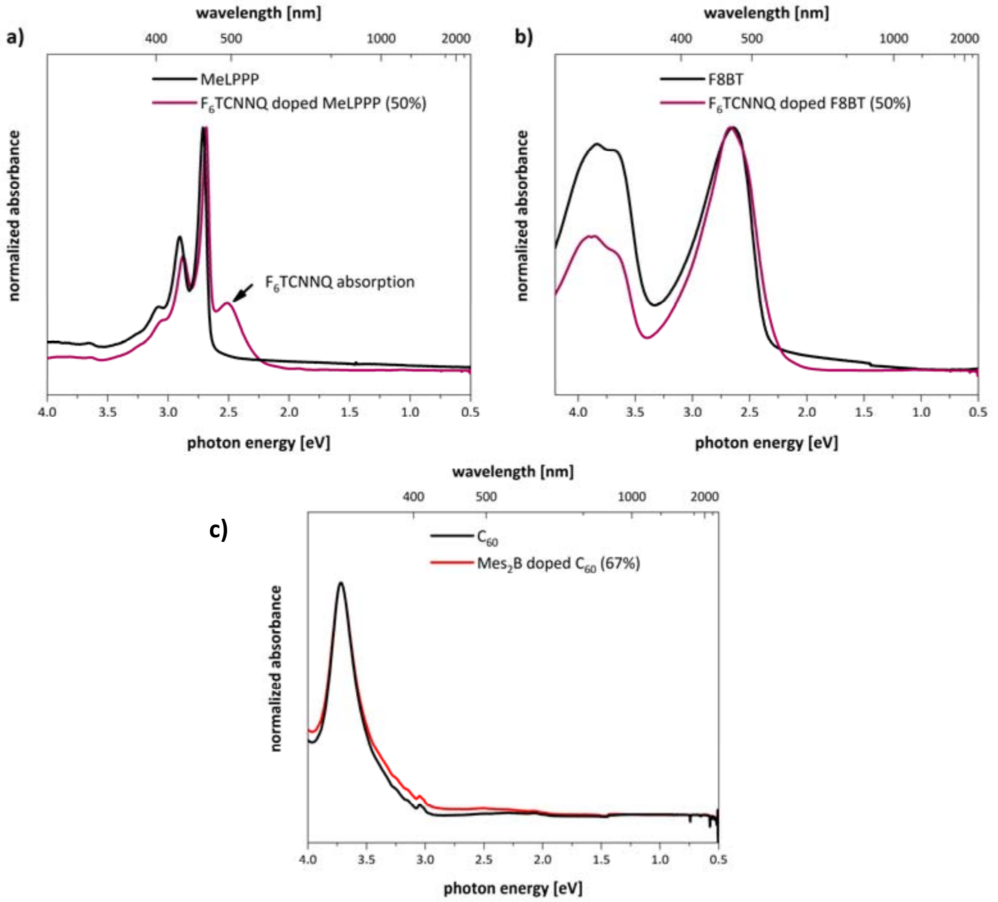


**Figure S12.** Optical absorption spectra of $F_6$TCNNQ-doped **a)** MeLPPP and **b)** F8BT solutions, and **c)** of $Mes_2B^+[B(C_6F_5)_4]^-$ doped $C_{60}$ solution. All spectra are normalized to their highest absorbance peak.

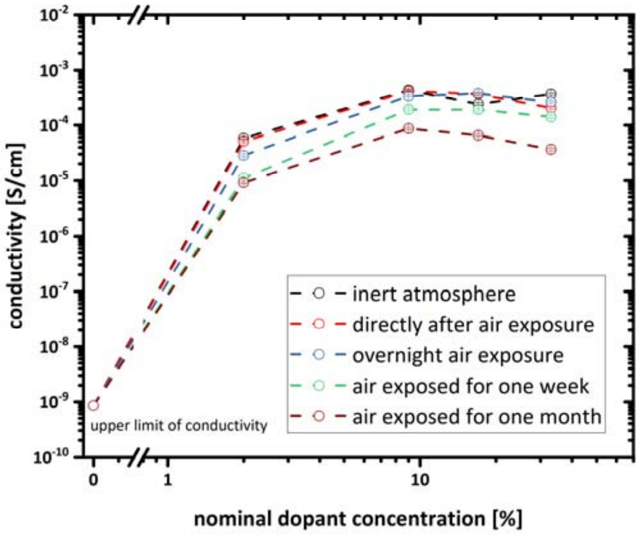


**Figure S13.** Conductivity measurements of $Mes_2B^+[B(C_6F_5)_4]^-$ doped MeLPPP films in inert atmosphere (black), after air exposure (red), after long time air exposure overnight (blue), for one week (green) and for one month (brown).

**Table S1.** Nominal dopant concentrations of the $Mes_2B^+[B(C_6F_5)_4]^-$ doped P3HT samples as well as concentrations estimated from the F/S area ratios from XPS and the average amount of charge per thiophene unit based on the hexamer model. All values in %.

| nominal | based on F/S area ratio | based on hexamer unit |
|---|---|---|
| 0.2 | 0.7 | 0.6 |
| 1 | 1.2 | 1.4 |
| 2 | 2.0 | 2.4 |
| 5 | 3.3 | 3.1 |
| 10 | 6.9 | 9.3 |
| 15 | 12.3 | 13.2 |
| 20 | 15.7 | 14.9 |
| 25 | 22.0 | 21.3 |
| 35 | 26.2 | 23.2 |

**Discussion: Shake-up transitions in core level spectra**

Another hypothesis regarding the change in the spectral shape of the S2p (and S2s) core levels is the possibility of the observed components at higher binding energy in the S2p (and S2s) core level to stem from enhanced shake-up transitions, like it was suggested for the $ClO_4^-$ doping of P3MT.[4] There, the authors found a similar line shape for the S2p (and in their case also C1s) core level as shown in Figure 5b and interpreted the asymmetrical line shape in terms of π-π* shake-up transitions, which are enhanced in intensity and also shifted in binding energy upon doping. In order to discuss this hypothesis, first the shake-up transitions of S2p and C1s in pristine P3HT are compared and then the C1s spectrum of the 35% $Mes_2B^+[B(C_6F_5)_4]^-$-doped P3HT sample is deconvoluted. For pristine P3HT, the π-π* shake-up transition for the S2p core level is located at around 2.6 eV to higher binding energies than the $S2p_{3/2}$ line position and its area is around 1.8% of the area of the S2p doublet, as shown in the deconvoluted S2p spectrum in Fig. S14a. The energy distance between the shake-up line and the main $S2p_{3/2}$ line is very similar to the transport gap of P3HT, reported to be 2.6 eV.[5] The deconvolution of the C1s spectrum of pristine P3HT in Fig. S14b shows a main contribution at 285.3 eV attributed to single- and double-bonded carbon species (C-C, C=C) in the 3HT unit and a smaller feature reduced in intensity by a factor of four relative to the C-C/C=C contribution. This smaller feature is shifted to higher binding energies by 490 meV and attributed to carbon nuclei bound to the sulfur atom in the thiophene unit (C-S), similar to what has been reported for P3HT before.[6] In addition, there is also a small contribution at 284.6 eV, which was attributed to adventitious carbon contaminations. The shake-up line was found at around 2.4 eV higher binding energy than the main C-C line and its area is around 2.3% of the combined C-C and C-S area, similar to the shake-up line of S2p.

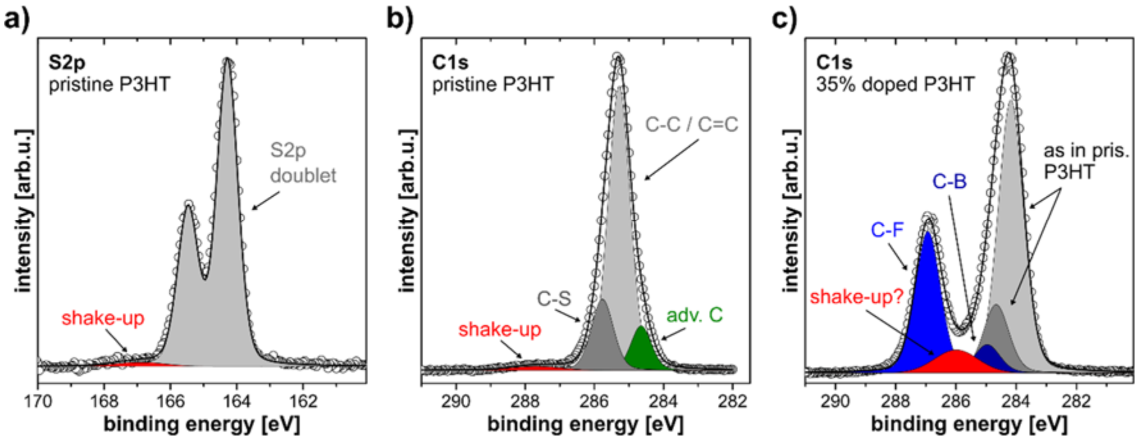


**Figure S14.** Deconvolution of XPS core level spectra taking into account shake-up lines for **a)** S2p and **b)** C1s of pristine P3HT as well as **c)** C1s of 35% $Mes_2B^+[B(C_6F_5)_4]^-$ doped P3HT. Circles display the raw data.

Assuming that the changed line shape upon doping results only from an enhancement and energetic shift of the shake-up contribution, the S2p spectrum of the 35% $Mes_2B^+[B(C_6F_5)_4]^-$ doped P3HT film (Figure 5b) would be fitted with only a S2p doublet and a broad peak for the assumed shake-up line. The shake-up transition would then be located at ca. 1.7 eV higher binding energy and its area could correspond to 37% of the doublet area. Proceeding similarly, the C1s spectrum of 35% doped P3HT can be fitted by five contributions, as shown in Fig. S14c. The main contribution stems from C-C and C-S species similar to pristine P3HT, while the prominent feature at higher binding energy stems from fluorine bonded species (C-F) from $[B(C_6F_5)_4]^-$. Here, the C-F contribution was linked to the amount of fluorine determined from the F1s core level in a ratio as found for $Mes_2B^+[B(C_6F_5)_4]^-$ multilayers. Another contribution of $[B(C_6F_5)_4]^-$ are boron bonded carbon species (C-B), which have an intensity ratio of 1:5 compared to the C-F contribution and are located at around 2 eV to lower binding energy as determined from the $Mes_2B^+[B(C_6F_5)_4]^-$ multilayers. The last contribution to the spectrum in Fig. S14c, is located at 1.9 eV higher binding energy than the C-C contribution and its area is around

12% of the combined C-S and C-C area. These results show, that if the additional contributions to the S2p and C1s line shape would stem from enhanced shake-up contributions, the enhancement of the S2p shake-up line by a factor of around 20 would be much stronger than the enhancement of the C1s shake-up line by a factor of around 5. This is considered to be highly unlikely, because there is no obvious reason for the shake-up transition (which for pure P3HT is in the same order of magnitude) to be enhanced more for the S2p level compared than for the C1s level. It is thus concluded that the asymmetry of the line shape of S2p as well as C1s towards higher binding energies observed in this study stems from polaronic and bipolaronic contributions. The reason for the smaller contribution of the C1s level compared to the S2p level lies in the fact that for C1s only the four carbon atoms in the aromatic ring are affected by the formation of polarons and bipolarons, while the six carbon atoms in the alkyl chain should be nearly unaffected by the formation.[7] Thus, they should lie at the same position as neutral C-C and contribute to the prominent feature at around 284 eV in the spectrum in Fig. S14c.

**Supporting Information References**


[1] A. Aguirre, P. Gast, S. Orlinskii, I. Akimoto, E. J. J. Groenen, H. El Mkami, E. Goovaerts, S. Van Doorslaer, *Phys. Chem. Chem. Phys.* **2008**, *10*, 7129.

[2] J. Niklas, K. L. Mardis, B. P. Banks, G. M. Grooms, A. Sperlich, V. Dyakonov, S. Beaupré, M. Leclerc, T. Xu, L. Yu, O. G. Poluektov, *Phys. Chem. Chem. Phys.* **2013**, *15*, 9562.

[3] J. F. Moulder, J. Chastain, *Handbook of X Ray Photoelectron Spectroscopy: A Reference Book of Standard Spectra for Identification and Interpretation of XPS Data*, Physical Electronics Division, Perkin-Elmer Corporation, Eden Prairie, **1995**.

[4] Y. Jugnet, G. Tourillon, T. M. Duc, *Phys. Rev. Lett.* **1986**, *56*, 1862.

[5] C. Deibel, D. Mack, J. Gorenflot, A. Schöll, S. Krause, F. Reinert, D. Rauh, V. Dyakonov, *Phys. Rev. B* **2010**, *81*, 085202.

[6] A. Lachkar, A. Selmani, E. Sacher, *Synth. Met.* **1995**, *72*, 73.

[7] R. Lazzaroni, M. Lögdlund, S. Stafström, W. R. Salaneck, J. L. Brédas, *J. Chem. Phys.* **1990**, *93*, 4433.
